# Regional years of life lost, years lived with disability, and disability-adjusted life-years for severe mental disorders in Guangdong Province, China: a real-world longitudinal study

**DOI:** 10.1186/s41256-022-00253-3

**Published:** 2022-06-20

**Authors:** Wenyan Tan, Lichang Chen, Yuqin Zhang, Junyan Xi, Yuantao Hao, Fujun Jia, Brian J. Hall, Jing Gu, Shibin Wang, Haicheng Lin, Xiao Lin

**Affiliations:** 1 Guangdong Mental Health Center, Guangdong Provincial People’s Hospital, Guangdong Academy of Medical Sciences, Guangzhou, Guangdong China; 2 Department of Medical Statistics and Center for Health Information Research and Guangdong Key Laboratory of Medicine, School of Public Health, Sun Yat-Sen University, 74 Zhongshan 2nd Rd, Guangzhou, 510080 Guangdong China; 3 Peking University Center for Public Health and Epidemic Preparedness & Response, Peking University, Beijing, 100191 China; 4 Global Public Health, New York University (Shanghai), Shanghai, 200122 China; 5 Sun Yat-Sen Global Health Institute, Sun Yat-Sen University, Guangzhou, 510080 Guangdong China

**Keywords:** Burden of disease, Disability-adjusted life-years, Severe mental disorders, Comorbidity, Socio-demographic index

## Abstract

**Background:**

To understand the magnitude and spatial–temporal distribution of the regional burden attributable to severe mental disorders is of great essential and high policy relevance. The study aimed to address the burden of severe mental disorders by evaluating the years of life lost, years lived with disability, and disability-adjusted life-years (DALYs) in Guangdong, China.

**Methods:**

We undertook a longitudinal study based on a multicenter database established by the Health Commission of Guangdong, involving a total of 21 prefectures and four economic regions in the Guangdong province. A total of 520,731 medical records from patients with severe mental disorders were collected for 2010–2020. Data were analyzed via an integrated evaluation framework by synthesizing prevalence estimates, epidemiological adjustment as well as comorbidity assessment to develop internally consistent estimates of DALY. DALY changes during 2010–2020 were decomposed by population growth and aging and further grouped by Socio-demographic Index (SDI). DALYs were projected to 2030 by the weighted median annualized rate of change in 2010–2020.

**Results:**

In 2010–2020, the average DALYs for severe mental disorders reached 798,474 (95% uncertainty interval [UI]: 536,280–1,270,465) person-years (52.2% for males, and 47.8% for females). Severe mental disorders led to a great amount of disease burden, especially in Guangzhou, Shenzhen, and Foshan cities. Schizophrenia and mental retardation with mental disorders were the two leading sources of the burden ascribed to severe mental disorders. Population growth and aging could be accountable for the increasing burden of severe mental disorders. Economic regions with higher SDI carried a greater burden but had lower annualized rates of change in DALYs. The overall burden of severe mental disorders is projected to rise modestly over the next decade.

**Conclusions:**

The findings urge prioritization of initiatives focused on public mental health, prevention strategies, health resources reallocation, and active involvement of authorities to effectively address the anticipated needs.

**Supplementary Information:**

The online version contains supplementary material available at 10.1186/s41256-022-00253-3.

## Background

Over the past three decades, China’s rapid economic growth was accompanied by an epidemiological transition from communicable to non-communicable diseases [1, 2]. Among the non-communicable diseases, mental disorders in China have historically been a low priority compared with cancers and cardiovascular diseases. Specifically, resource allocation, both financial and human, for mental disorders has been much scarce due to the inadequate understanding of public health policies and insufficient design of the healthcare delivery system [3, 4]. However, mental disorders are one of the primary sources of disease burden for population health [5, 6], leading to ongoing direct and indirect treatment costs [7, 8]. Previous research demonstrated that the lifetime prevalence of mental disorders in China may have reached 15.8–16.6% [9, 10], whereas the average treatment-seeking rate is only less than 6% [11, 12]. The severe gaps between detection and treatment of mental disorders may partly be ascribed to the incomplete knowledge about the magnitude of losses of healthy life-years in patients before resource allocation for diagnosis, treatment as well as management of these disorders [7]. Therefore, a better understanding of the magnitude of the disease burden attributed to mental disorders is crucial to prioritize the management of these conditions.

Findings from the Global Burden of Disease (GBD) study have resulted in a paradigm shift from determining the health loss due to mental disorders by prevalence rates, to conceptualizing such losses based on disability-adjusted life-years (DALYs) [7, 13]. The GBD 2017 reported the years of life lost (YLLs), years lived with disability (YLDs), and DALYs ascribed to mental health disorders in China’s 34 provinces as well as special administrative regions [2]. Mental health disorders ranked second place for non-fatal health outcomes measured by YLDs, and severe mental disorders such as schizophrenia are the leading causes of DALYs. Among the 34 provinces, Guangdong ranked first place for DALYs attributable to mental health disorders, with an estimated DALY of 1.84 (95% uncertainty interval [UI]: 1.38–2.37) million person-years by 2017. Given limited health resources as well as current management and intervention programs, the Chinese healthcare delivery system focused predominantly on severe mental disorders such as schizophrenia, schizoaffective disorder, mental retardation with mental disorders, and so forth [14–16]. Due to the rapid economic development, aging population as well as social-economic disparities in Guangdong [17, 18], there is a pressing need to quantify the regional spatial–temporal trends of DALYs for severe mental disorders to support evidence-based decision-making in public mental health. However, a systematic quantification of the magnitude as well as spatial–temporal distribution for regional burden attributable to severe mental disorders in Guangdong is not readily available.

Since 2010, Guangdong Health Commission has established the Guangdong Mental Health System (GDMHS), a population-based surveillance network for collecting the real-world medical records of such patients [18]. Data were gathered through efforts of consistent methodology including coherent diagnostic nomenclature, fully structured diagnostic interviews, and sophisticated household surveys [19, 20]. In this study, using the well-established GDMHS, we aim to estimate the regional disease burden associated with severe mental disorders in Guangdong and to present a detailed account of changes in the burden due to mental disorders, from 2010 to 2020. We also aim to project the estimated burden up to 2030, to inform prevention strategies as well as relevant policy-making to reduce the burden attributable to mental disorders.

## Methods

### Study design and population

This longitudinal study was conducted to evaluate the regional disease burden associated with severe mental disorders using mental health surveillance records from GDMHS. Within the GDMHS platform, the longitudinal data concerning information on diagnosis, medication, risk of violent behavior, etc*.*, were collected and maintained in response to the National Health Commission of China [21], and it has extensive coverage of all of the 21 prefecture cities in Guangdong province. The province is located in southern China and adjacent to the Hong Kong and Macao Special Administrative Region. It remains the largest economic body in China and has a high population density of 115.21 million permanent residents [22]. According to the gross domestic product by prefecture, Guangzhou, Shenzhen, and Foshan are recognized as the most economically advantaged cities among the 21 prefectures. These prefectures were further categorized into four economic regions, namely, the Greater Bay Area, the North region, the West region, and the East region. The study complies with the Guidelines for Accurate and Transparent Health Estimates Reporting (GATHER) statement.

### Covariates

Variables available on the GDMHS platform included: (a) de-identified individual-level demographic information such as the residential address, age, biological gender, education level, marital and employment status; (b) a follow-up medical record of diagnosis for mental disorders and relevant comorbidities (shown in Additional file 1: Table S1); (c) family and genetic history of diagnosed mental disorders; and d) survival status (coded in the 10th revision of the International Classification of Diseases [23], ICD-10). Information on extracted variables was summarized in Table 1.

**Table 1:** Descriptive statistics of the collected study records (N = 520,731)

Variables	N (%)
*Age group (years)*	
< 7	3347 (0.6)
7–12	11,144 (2.1)
13–17	20,823 (4.0)
18–44	285,383 (54.8)
45–64	167,124 (32.1)
≥ 65	32,910 (6.3)
*Sex*	
Female	229,448 (44.1)
Male	291,283 (55.9)
*Residence*	
Rural	355,988 (68.4)
Urban	164,743 (31.6)
*Educational level*	
Illiteracy	115,532 (22.2)
Elementary school	173,995 (33.4)
Junior high school	141,119 (27.1)
Senior high school or specialized secondary school	46,246 (8.9)
Tertiary school or university or higher	17,367 (3.3)
Others	26,472 (5.1)
*Marital status*	
Unmarried	241,045 (46.3)
Married	232,169 (44.6)
Divorced	20,319 (3.9)
Widowed	15,152 (2.9)
Unspecified	12,046 (2.3)
*Employment status*	
Unemployed	513,609 (98.6)
Employed^a^	7106 (1.4)
Unspecified	16 (0.0)
*Economical level*	
Poverty^b^	246,819 (47.4)
Non-poverty	273,912 (52.6)
*Mental disorders by ICD*^*c*^* groups*	
F00-F09: Organic, including symptomatic, mental disorders	29,384 (5.6)
F10-F19: Mental and behavioral disorders due to psychoactive substance use	3485 (0.7)
F20-F29: Schizophrenia, schizotypal and delusional disorders	333,366 (64.0)
F30-F39: Mood [affective] disorders	46,681 (9.0)
F40-F48: Neurotic, stress-related, and somatoform disorders	752 (0.1)
F50-F59: Behavioral syndromes associated with physiological disturbances and physical factors	112 (0.0)
F60-F69: Disorders of adult personality and behavior	125 (0.0)
F70-F79: Mental retardation (Intellectual disabilities) ^d^	105,959 (20.3)
F80-F89: Disorders of psychological development	251 (0.0)
F90-F98, F99: Behavioral and emotional disorders with onset usually occurring in childhood and adolescence, and unspecified mental disorder	616 (0.1)
*Family history*	
No	468,800 (90.0)
Yes	31,406 (6.0)
Unspecified	20,525 (3.9)
*Screening for genetic history*	
No	512,533 (98.4)
Yes	8067 (1.5)
Unspecified	131 (0.0)
*Deceased*^*e*^	9298 (1.8)
Cerebrovascular diseases (I60-I69)	1203 (12.9)
Cardiovascular diseases (I20-I25, I26-I51)	1053 (11.3)
Diseases of the respiratory system (J00-J98)	599 (6.4)
Neoplasms (C00-D48)	499 (5.4)
Diseases of the digestive system (K00-K92)	245 (2.6)
Other causes (excluding psychotic disorders)	5699 (61.3)

^a^Employed workers include professional technology personnel; clerks and relevant personnel; manufacturing and related personnel; agriculture, forestry, husbandry and fishery production, and auxiliary personnel, etc

^b^Poverty is defined as the residents and households living under the urban and rural subsistence allowance

^c^ICD: World Health Organization International Classification of Diseases, 10th revision

^d^According to the work specification for management and treatment of severe mental disorders (2018 edition) [21], severe mental patients with mental retardation are referred to as patients having mental retardation with mental disorders in the Chinese context

^e^Mental patients in the study were provided with follow-up services by local community health centers, from which death information regarding the causes of death was extracted. Causes of death were also coded according to ICD-10

### Data collection and processing

We collected valid registered medical records from GDMHS, covering January 1, 2010, to December 24, 2020. From the surveillance-based system, de-identified medical information was extracted on mental health disorders diagnosed by ICD-10 codes (F00-F99). Each record from GDMHS was validated and confirmed by the Health Commission of Guangdong upon data entry [2, 9]. The registered medical records consisted of 520,731 individuals that were clinically diagnosed with severe mental disorders including schizophrenia, schizoaffective disorder, paranoid psychosis, bipolar disorder, psychotic disorder due to epilepsy, and mental retardation with mental disorders [3, 24]. These disorders were anchored according to the lawful definition of severe mental disorders in the Chinese context [14, 25], and more details concerning information from GDMHS can be found in Additional file 1: Figure S1 and elsewhere [18]. Our collected dataset did not contain personally identifiable information and individuals could not be contacted, and thereby individual informed consent was not required. For data pre-processing, repeated records within the same year were treated as one single record, given the need for annual estimates of the burden metrics [26]. We collected city-specific population data and relevant information from the Guangdong Statistical Yearbook (2010–2020) [22] and the Global Health Data Exchange site of the Institute for Health Metrics and Evaluation. [27]

### Data analysis

#### Burden of disease estimation

DALYs are calculated as the sum of YLL due to premature mortality and YLD due to non-fatal disability, thus incorporating both mortality and morbidity information. Using the DALY metric, the burden of disease across different cities with varying population structures can be directly compared [28–30]. We also presented age-standardized DALYs, which allowed comparisons over time and across different areas. The age-standardized rates were based on the Chinese population reported by the GBD 2019. [31]

As indicated by previous research [28, 32, 33], YLDs are the main contributors to DALYs attributed to mental disorders. In the study, underlying causes of death from the database did not register mental disorders as direct causes of death (Table 1). Thus, we only estimated prevalence from the collected records. Next, YLDs were estimated by multiplying the prevalence cases by disability weight (DW) [2, 33]. DW quantifies the magnitude of health loss associated with mental disorder on a scale from 0 (perfect health) to 1 (equivalent to death) and was extracted from the latest GBD (Additional file 1: Table S1) [29]. YLDs attributed to each mental disorder were then computed by applying the age-sex-city-year-specific cause distribution to the estimates [34]. For YLL, we used the indirect method by applying the estimated YLDs to the YLL/YLD ratios. This method was often used in the burden of disease study where information related to burden estimates was unavailable [35, 36]. The YLL/YLD ratios were summarized in Additional file 1: Table S2.

#### Prevalence estimates and epidemiological adjustment

To represent the continuous survival status of patients, and to better reflect the years lived with non-fatal mental disorders, incident counts of newly-diagnosed cases from the follow-up records were accumulated over years to generate crude prevalence counts by age, sex, and prefecture city. Deceased patients were not included in the estimation of crude prevalence. Age-sex-city-specific crude prevalence was calculated as the ratio between the annual accumulated number of diagnosed patients and the population in each city.

Next, to generate internally consistent epidemiological estimates, the age-sex-city-specific prevalence was adjusted using an epidemiological system dynamic model similar to the GBD method [29, 37]. A two-compartment framework with susceptible (*S*) and condition (*C*) compartments was implemented to account for the process of incidence, remission, and mortality. Based on the estimated number of patients with the condition and the number of susceptible populations, the adjusted age-sex-city-specific prevalence of mental disorders was calculated. Further information describing the epidemiological adjustment can be found in the appendix (Additional file 1: Table S12).

#### Comorbidity assessment

In line with the GBD study, we applied comorbidity assessment to the disability weight of mental disorders in the calculation of YLDs [29, 38]. Under the analysis framework, we performed comorbidity simulations separately for age-sex-city-specific estimates to account for the comorbidities amongst individuals. We synthesized a population of 20,000 individuals ($n$) and exposed the synthetic population to the binomial probability of having all the different comorbidities recorded in the GDMHS database. Using the synthesized population, a total of 11 comorbidities extracted from the follow-up records (Additional file 1: Table S1) were considered in the comorbidity assessment. Comorbidity-adjusted DW, as well as comorbidity-adjusted YLD rates attributed to severe mental disorders, were calculated, yielding the age-sex-city-year-specific YLD estimates. Next, we reported 95% uncertainty intervals (UI) by conducting a bootstrap analysis with 1000 repetitions, after which 95% uncertainty intervals were generated as the 2.5^th^ and 97.5^th^ values of the posterior distribution.

#### Decomposition and projection of DALY over time

To explore the underlying reasons for changes in disease burden over time, we implemented the time decomposition method proposed by Charlson [7] for DALY ascribed to mental health disorders. A total of two scenarios were set, with one to account for population growth and the other to explain the influence of population aging. Further details were summarized in the appendix. In the population growth scenario, the percentage changes between DALYs and scenario expected values reflect the change in absolute numbers due to population growth. The percentage change for the second scenario represents the change in absolute numbers due to changing age structure.

As a final step, we projected DALYs ascribed to mental disorders for each region of Guangdong to 2030, while implementing methods of the weighted median annualized rate of change [39, 40]. The projection process was summarized in the appendix methods and Additional file 1: Table S13. To be consistent with the GBD estimates, weighted median annualized rates of change in DALYs were categorized by the Socio-demographic Index (SDI), a composite indicator of development status ranging from 0 to 1 [31]. Details on SDI can be found elsewhere [41]. Five SDI quintiles (high, high-middle, middle, low-middle, and low SDI state groups) were empirically calculated. We conducted an exploratory analysis of the associations between the SDI and disease burden indicator, for logarithm and annualized rate of change of DALYs, based on Pearson correlation. Analyses were performed in R (version 3.6) and Python (version 3.8). A *p*-value of less than 0.05 was considered to be statistically significant.

## Results

Table 1 shows the summary descriptive statistics in the study. From January 1, 2010, to December 24, 2020, records for a total of 520,731 individuals that were clinically diagnosed with severe mental disorders in Guangdong were extracted from GDMHS. The mean (SD) age at study enrollment was 39.7 (15.7) years, and most patients were aged 18 ~ 44 (54.8%). A total number of 229,448 (44.1%) individuals were females, while 291,283 (55.9%) were males. Sex-specific and residence-specific summary descriptive statistics were further depicted in Additional file 1: Table S3.

### DALY of severe mental disorders

For overall DALYs of severe mental disorders, the average number of DALYs in 2010–2020 was estimated to be 798,474 (95% UI: 536,280, 1,270,465), of which 52.2% were estimated in males. We also estimated DALYs for mental disorders without comorbidity adjustment, which would shrink down to a number of 274,789 (95%UI: 191,823, 359,284) person-years. Table 2 demonstrates that the top three prefectures whose comorbidity-adjusted DALYs ranked the most were Guangzhou (183,090 with 95% UI: 122,811, 292,087), Shenzhen (150,599 with 95% UI: 100,871, 240,217), and Foshan (122,803 with 95% UI: 82,277, 195,698). Additional file 1: Table S4 presented the DALY estimates by urban and rural residents. Though the ranking of DALY burden by residence remains largely the same as the overall ranking, the number of DALY of severe mental disorders was significantly higher in rural residents across a few cities in the non-Greater Bay Area (namely, Zhanjiang, Qingyuan, Meizhou, Jieyang, Shaoguan, Maoming, Heyuan, and Yunfu), with non-overlapping 95% uncertainty intervals. Decomposition of DALYs into YLL and YLD by city, sex, and residence was shown in Additional file 1: Tables S5–S6.

**Table 2:** Average disability-adjusted life-years (DALYs) for patients with severe mental disorders in Guangdong, China, during 2010–2020, by sex and prefectural city

Prefectural city	DALY (non-adjusted)		DALY (comorbidity-adjusted)		Age-standardized^a^ DALY rate (per 100,000, non-adjusted)		Age-standardized DALY rate (per 100,000, comorbidity-adjusted)	
	Male (95% UI^b^)	Female (95% UI)	Male (95% UI)	Female (95% UI)	Male (95% UI)	Female (95% UI)	Male (95% UI)	Female (95% UI)
Guangzhou	14,009 (9778, 18,319)	11,053 (7715, 14,452)	95,476 (64,050, 152,329)	87,614 (58,761, 139,758)	205.18 (143.22, 268.26)	179.36 (125.21, 234.48)	1373.68 (907.87, 2206.72)	1387.28 (915.68, 2226.43)
Shenzhen	7607 (5310, 9946)	6031 (4210, 7885)	78,575 (52,637, 125,355)	72,024 (48,234, 114,862)	127.64 (89.10, 166.88)	112.00 (78.18, 146.42)	1345.71 (887.80, 2160.80)	1359.05 (895.46, 2179.91)
Foshan	10,357 (7230, 13,543)	8204 (5727, 10,727)	64,041 (42,911, 102,069)	58,762 (39,366, 93,629)	277.91 (193.99, 363.38)	243.86 (170.23, 318.84)	1667.08 (1100.58, 2673.64)	1683.69 (1110.20, 2697.40)
Dongguan	8822 (6158, 11,534)	6983 (4875, 9130)	29,105 (20,740, 43,828)	26,138 (18,508, 39,608)	218.79 (152.73, 286.08)	191.98 (134.02, 251.01)	697.43 (492.23, 1054.06)	689.66 (483.25, 1048.12)
Zhanjiang	14,855 (10,369, 19,424)	11,780 (8223, 15,402)	24,854 (16,622, 39,696)	22,790 (15,237, 36,386)	422.83 (295.14, 552.88)	371.35 (259.21, 485.54)	680.92 (448.65, 1094.28)	687.65 (452.52, 1103.80)
Zhuhai	1671 (1167, 2185)	1325 (926, 1733)	16,819 (11,290, 26,827)	15,416 (10,345, 24,580)	190.96 (133.39, 249.67)	167.62 (117.09, 219.15)	1927.19 (1274.46, 3092.34)	1943.97 (1283.80, 3115.55)
Qingyuan	6548 (4572, 8562)	5192 (3624, 6787)	14,691 (9825, 23,425)	13,497 (9022, 21,511)	352.76 (246.27, 461.21)	309.73 (216.23, 404.92)	757.42 (499.61, 1214.35)	764.31 (503.44, 1223.85)
Meizhou	8404 (5866, 10,987)	6655 (4645, 8701)	13,837 (9249, 22,137)	12,675 (8468, 20,275)	400.18 (279.32, 523.21)	351.28 (245.20, 459.24)	631.84 (416.17, 1016.90)	637.18 (419.12, 1024.50)
Jiangmen	9385 (6551, 12,271)	7426 (5184, 9709)	10,847 (7241, 17,358)	9948 (6638, 15,913)	426.31 (297.59, 557.42)	374.03 (261.11, 489.03)	472.10 (310.69, 759.81)	476.59 (313.22, 766.15)
Huizhou	7333 (5119, 9587)	5802 (4050, 7585)	9251 (6158, 14,815)	8481 (5645, 13,580)	317.35 (221.52, 414.90)	278.37 (194.32, 363.91)	383.83 (251.91, 618.33)	387.38 (253.93, 623.38)
Jieyang	7364 (5141, 9629)	5842 (4078, 7638)	8603 (5703, 13,804)	7889 (5228, 12,655)	251.78 (175.76, 329.22)	221.20 (154.42, 289.21)	281.55 (184.04, 454.31)	284.27 (185.56, 458.16)
Shaoguan	6825 (4764, 8924)	5398 (3768, 7057)	7895 (5273, 12,628)	7236 (4831, 11,572)	477.26 (333.15, 624.01)	418.59 (292.21, 547.26)	530.27 (349.03, 853.56)	535.26 (351.80, 860.76)
Zhongshan	2646 (1847, 3459)	2094 (1461, 2737)	6904 (4601, 11,059)	6323 (4211, 10,126)	167.57 (116.96, 219.05)	146.95 (102.58, 192.08)	422.30 (277.40, 680.28)	425.77 (279.28, 685.13)
Maoming	13,131 (9165, 17,170)	10,396 (7257, 13,593)	6142 (4051, 9900)	5634 (3714, 9077)	438.04 (305.76, 572.79)	384.28 (268.24, 502.46)	197.45 (128.40, 320.32)	199.22 (129.35, 322.82)
Heyuan	5110 (3568, 6682)	4051 (2829, 5297)	6023 (4011, 9646)	5518 (3673, 8836)	344.12 (240.27, 450.02)	302.20 (211.01, 395.18)	389.75 (255.93, 628.29)	393.06 (257.76, 633.01)
Shantou	4824 (3368, 6306)	3832 (2676, 5010)	5915 (3897, 9555)	5414 (3565, 8743)	178.62 (124.70, 233.51)	157.07 (109.66, 205.33)	211.30 (137.18, 343.42)	212.96 (138.05, 345.78)
Zhaoqing	8127 (5673, 10,625)	6431 (4489, 8408)	5713 (3794, 9175)	5229 (3471, 8399)	410.32 (286.42, 536.47)	359.91 (251.24, 470.53)	277.80 (181.76, 448.70)	279.88 (182.81, 451.71)
Yangjiang	5214 (3640, 6818)	4132 (2885, 5403)	4033 (2677, 6467)	3698 (2454, 5929)	426.41 (297.71, 557.60)	374.40 (261.41, 489.55)	316.67 (207.20, 510.86)	319.69 (208.90, 515.31)
Yunfu	5561 (3882, 7271)	4407 (3077, 5761)	3224 (2136, 5176)	2958 (1959, 4747)	461.62 (322.26, 603.52)	405.27 (282.93, 529.81)	257.09 (168.05, 414.90)	259.53 (169.41, 418.35)
Chaozhou	2413 (1684, 3154)	1911 (1334, 2499)	2830 (1862, 4572)	2592 (1705, 4187)	187.76 (131.07, 245.47)	164.81 (115.05, 215.46)	209.96 (136.23, 341.53)	211.64 (137.10, 343.93)
Shanwei	3142 (2195, 4110)	2496 (1743, 3264)	2016 (1315, 3275)	1844 (1202, 2996)	217.44 (151.85, 284.35)	191.14 (133.49, 249.95)	133.48 (85.83, 218.22)	134.50 (86.34, 219.76)

^a^Estimated values for age-standardized rates were calculated based on the Chinese population reported by the GBD 2019 [31]

^b^UI: uncertainty interval

### Trends and patterns from 2010 to 2020

Table 3 shows the changes in DALY absolute numbers and age-standardized rates between 2010 and 2020 for four economic regions of Guangdong. For the overall changes, these regions have seen a dramatic increase in the DALY burden from 2010 to 2020. However, increases in DALYs may be accounted for by different scenarios. For instance, even though Greater Bay Area remained the top region with the highest DALYs during 2010–2020, the proportions of change in absolute numbers that were due to population growth (39.96%) and population aging (39.87%) were relatively smaller than those in the non-Greater Bay Area (ranging from 65.75% ~ 76.32%). Besides, standardization of DALY rates across four regions reflected the deteriorating burden of disease over 2010–2020, regardless of age-sex-specific structures, particularly for the non-Greater Bay Area. Sex-specific and residence-specific scenario decomposition were shown in Additional file 1: Tables S7–S10.

**Table 3:** Change in absolute DALY^a^ numbers and age-standardized^b^ rates (per 100,000) for patients with severe mental disorders in Guangdong, China, between 2010 and 2020, by economic region

Region	2010 DALYs (95% UI^c^)		2020 DALYs (95% UI)		Change in absolute numbers due to population growth (%)	Change in absolute numbers due to changing age structure (%)	Overall change 2010–2020 (%)^*^
	Numbers	Age-standardized rates (per 100,000)	Numbers	Age-standardized rates (per 100,000)			
Greater Bay Area^d^	361,861 (242,732, 577,665)	672.71 (443.25, 1083.01)	690,627 (467,967, 1,086,208)	1111.83 (744.26, 1756.92)	39.96	39.87	65.28
North Guangdong^e^	25,151 (16,785, 40,093)	191.94 (125.88, 308.49)	110,862 (74,568, 175,859)	798.00 (529.72, 1271.96)	76.32	76.29	315.75
West Guangdong^f^	27,233 (18,065, 43,488)	161.55 (105.22, 260.33)	85,177 (57,026, 135,743)	467.62 (309.25, 748.89)	65.79	65.75	189.47
East Guangdong^g^	12,678 (8411, 20,322)	78.76 (51.32, 127.29)	47,347 (31,451, 75,679)	281.32 (184.61, 451.70)	72.48	72.45	257.19

^a^DALY: disability-adjusted life-years, for which comorbidity-adjusted estimates were reported;

^b^Estimated values for age-standardized rates were calculated based on the Chinese population reported by the GBD 2019 [31]

^c^UI: uncertainty interval

^d^Greater Bay Area: including Guangzhou, Foshan, Zhaoqing, Shenzhen, Dongguan, Huizhou, Zhuhai, Zhongshan, Jiangmen

^e^North Guangdong region: referring to Shaoguan, Qingyuan, Meizhou, Heyuan

^f^West Guangdong region: referring to Zhanjiang, Yangjiang, Maoming, and Yunfu

^g^East Guangdong region: referring to Chaozhou, Shantou, Jieyang, and Shanwei

^*^DALY rates are age-standardized and sex-standardized so change in rates reflects changes in factors related to prevalence rates in addition to population growth and age structure

**Fig. 1:**
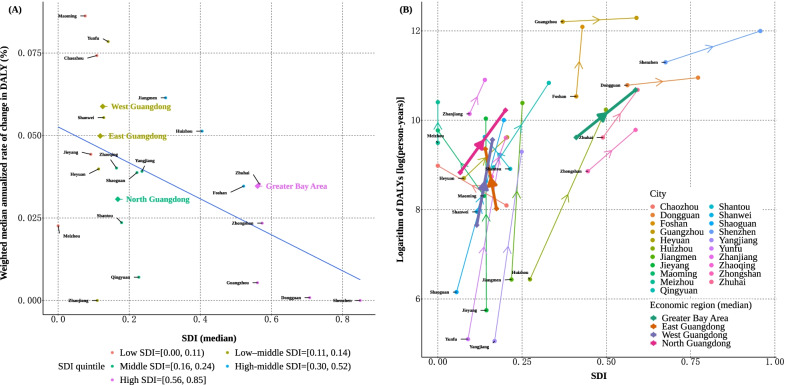
Weighted median annualized rates of change (**a**) and change of logarithm of values (**b**) in disability-adjusted life-years (DALYs) for patients with severe mental disorders in Guangdong, China, during 2010–2020, by SDI group. Notes: SDI = Socio-demographic Index. SDI groups were generated by calculating quintiles at the set of percentiles p = {0, 0.2, 0.4, 0.6, 0.8, 1.0}, in which five categories of SDI were yielded (namely, low SDI < 0.11, Low-middle SDI < 0.14, Middle SDI < 0.24, high-middle SDI < 0.52, and High SDI ≥ 0.56). In (**a**), the median of SDI during 2010–2020 was plotted, whereas the blue line reflects the linear relationship between estimated SDI and weighted median annualized rates of change in DALYs ascribed to severe mental disorders. In (**b**), the logarithm of DALYs across 21 prefecture cities was further aggregated by the median for the four economic regions. Arrows indicate the direction of change in the logarithm of DALY, where comorbidity-adjusted values were reported

Changing pattern of DALY burden was further illustrated in Fig. 1 (Fig. 1a for annualized rates of change by SDI quintile groups, and 1B for change of logarithm of DALYs). We found a modest negative correlation between SDI and annualized rates of change in DALYs ascribed to severe mental disorders across 21 prefectures and 4 economic regions of Guangdong (r = -0.49, *p* = 0.015). The East and West regions were similar in terms of SDI state groups. The logarithm of DALYs changed drastically for the non-Greater Bay Area from 2010 to 2020. Most prefectures in Greater Bay Area had higher SDIs, whereas prefecture cities in non-Greater Bay Areas clustered at low- to middle-SDI state groups. We also found a significant positive association of the estimated SDIs with the logarithm of DALYs across all prefectures (For 2010, r = 0.52, *p* = 0.009; for 2020, r = 0.62, *p* = 0.002). Regional and prefectural SDIs were presented in Additional file 1: Table S11. Similar patterns for the association of SDI with DALYs were observed for schizophrenia and mental retardation with mental disorders (Additional file 1: Figures S4–S5).

### Cause-specific averaged DALYs during 2010–2020

The averaged DALYs attributable to specific severe mental disorders were demonstrated in Additional file 1: Figure S3. The burden of severe mental disorders in the younger age groups under 20 years was primarily due to mental retardation with mental disorders, which accounted for around 17.0% (95% UI: 12.3, 24.4) of severe mental disorders for patients aged under 20 years. In contrast, the mental burden in patients in working-age groups (aged 20–69 years) was accounted for by schizophrenia, which took up around 12.4% (95% UI: 11.5, 13.7) for those aged 20–69 years. For all age groups combined, the absolute number and age-standardized rates of DALYs for schizophrenia were generally higher than those for mental retardation with mental disorders. The burden of schizophrenia cost an average number of 400,811 DALYs (95% UI: 271,132, 625,005) and 409.83 person-years per 100,000 population (95% UI: 272.40, 643.91), respectively, followed by mental retardation with mental disorders (with absolute number as well as age-standardized rates being 238,078 DALYs [95% UI: 157,498, 393844] and 211.39 person-years [95% UI: 139.21, 350.89] per 100,000, respectively).

### Regional patterns through 2030

**Fig. 2:**
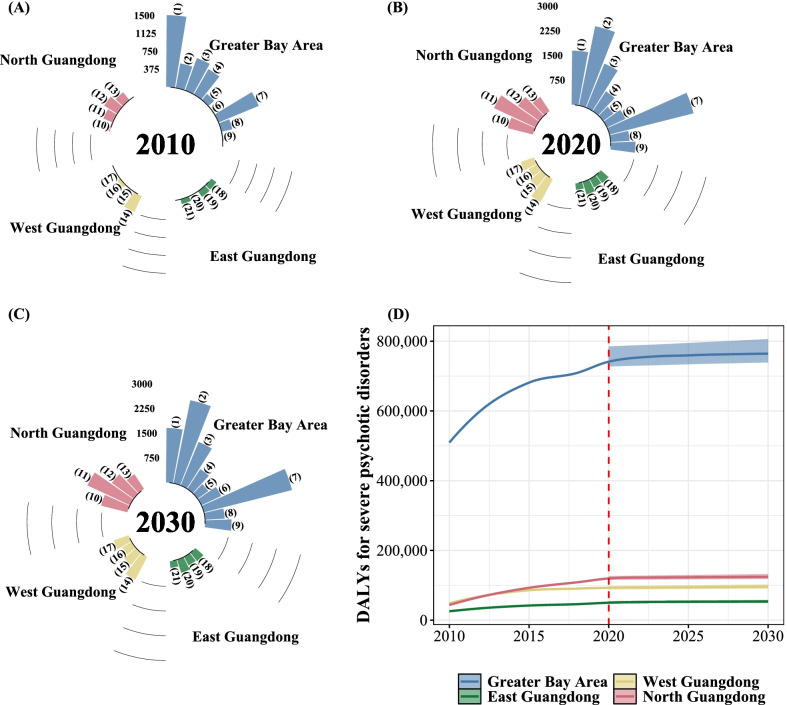
Temporal trends (**a–c**) for age-standardized DALY^a^ rates and forecasts (**d**) for absolute DALYs ascribed to severe mental disorders in Guangdong, China, during 2010–2030, by economic region. ^a^DALY: disability-adjusted life-years, for which comorbidity-adjusted estimates were reported. Age-standardized rates were calculated based on the Chinese population reported by the GBD 2019 [31]. Notes: In (**a–c**), region-specific patterns of age-standardized DALYs ascribed to severe mental disorders during 2010–2030 were depicted. The Greater Bay Area refers to Guangzhou(1), Foshan(2), Zhaoqing(5), Shenzhen(3), Dongguan(4), Huizhou(6), Zhuhai(7), Zhongshan(8), Jiangmen(9). The North Guangdong region refers to Shaoguan(10), Qingyuan(11), Meizhou(12), and Heyuan(13). The West Guangdong region refers to Zhanjiang(14), Yangjiang(15), Maoming(16) and Yunfu(17). Finally, the East Guangdong region refers to Chaozhou(18), Shantou(19), Jieyang(20), and Shanwei(21). In panel (D), temporal trends between 2010 and 2030 were shown, for four economic regions of Guangdong. Uncertainty intervals of forecasts for DALYs were presented in shaded colors

For the course of future burden in the next decade, the number of DALY attributed to severe mental disorders for the Greater Bay Area and North regions is expected to remain at a high level (Fig. 2), albeit the smaller annualized rates of change in DALY (0.035% and 0.031%, respectively for Greater Bay Area and North region). In the East and West regions, rising patterns were also observed in 2010–2020 but with slightly higher annualized rates of change (0.05% and 0.059%, respectively), leading to the increase of DALYs.

## Discussion

Severe mental disorders have become a major public health concern in China. This study reports for the first time the current size and future burden of severe mental disorders for the Guangdong province, the most populous province and the key economic contributor to China. Schizophrenia and mental retardation with mental disorders accounted for a great proportion of severe mental disorders, which consume most health resources and bring great economic loss to patients and society [18]. Overall DALYs attributable to severe mental disorders in Guangdong may increase in the coming decades, where the DALY burden for the Greater Bay Area remains the heaviest. While the shift to a much greater number of DALYs currently occurred in the working-age groups, with an oversize population, aging structure, and extension of life expectancy in the next decades [2], policy-makers should remain aware of how aging shapes future health needs and understand the importance of prioritizing resources for intervention, treatment, rehabilitation, and prevention of severe mental disorders. Indeed, the number of DALYs represents the burden of disease that the health systems in a region must face. To the best of our knowledge, this is the first regional burden of disease study implementing the standard DALY metric while also incorporating the integrated information from a large-scale population-based medical system in China.

Our findings reiterate the complexity of management and intervention programs for patients with severe mental disorders because these patients may have mental disorder comorbidity [42]. As shown in Table 1, mental disorders not belonging to the six types of severe mental disorders were also registered in the GDMHS database, including behavioral syndromes, stress-related disorders, disorders of psychological development, and so forth. Comorbidities of these mental disorders nevertheless contributed to the overall disease burden for patients and the health services system as a whole [43]. Although mechanisms about how each mental disorder interacts with one another are beyond the scope of the current paper, implications drawn from our study are well applicable to the medical treatment and rehabilitation of patients with severe mental disorders. Due to the dynamics of synergistically related social-economic determinants, biological conditions, as well as complex conditions of mental health patients [44], health professionals may need to keep track of patients’ mental disorder comorbidities that are manifested either in sequence or simultaneously, while tailoring treatment strategies for precise medicine therapies. Moreover, the development of innovative medical treatment and the constant need to evaluate comorbidity dynamics over time in patients with severe mental disorders may be highly relevant to establishing clinical training programs for mental health professionals. It is worth noting that, there remains a pressing need for the cultivation of a greater number of mental health professionals, as supported by our foresight of a steady increase in the overall burden of severe mental disorders in the next decade. The aggregated burden of severe mental disorders over years will greatly increase the needs and demands for mental health services in the population [45]. However, as suggested by previous research [7, 46, 47], the shortage of human resources in public mental health for the Chinese population would not see improvement if training programs for mental health professionals and policy initiatives for mental health care remain unreformed. Active involvement of authorities in public mental health may also help remove barriers to accessing equitable and high-quality mental health services for the public [46]. Thereby, expanding the mental health workforce with qualified training programs, and higher involvement of authorities are the key priorities for public mental health in the coming decades in China.

DALYs ascribed to severe mental disorders that manifest predominantly during adulthood were generally higher in the high-SDI quintile group than in the low- and middle-SDI quintiles, which is consistent with GBD studies [13]. We found that the estimated SDI states were positively correlated with the DALY burden, while negatively correlated with the annualized rates of change in DALY. These relations reflect the association between education, income, as well as fertility level of a specific area, and the DALY indicator. As previously reported [18, 48, 49], lack of education and poverty are well-known determinants for mental disorders. Within the Greater Bay Area, Guangzhou, Shenzhen, and Foshan are more economically-empowered [22], the medical conditions and health services are therefore more readily available. As stated by Tan and colleagues [18], severe mental disorders in economically-developed cities are better managed and treated. We suspect that this may account for the lower annualized rates of change among some economically-empowered cities. However, due to the higher population density and an oversize population (illustrated in Additional file 1: Figure S1) [10, 50], the absolute numbers of DALYs in the high-SDI state group are notable, indicating the historically high burden in the Greater Bay Area.

As for the less developed regions, such as the East and West regions, even though the population density is lower, their smaller SDI quintiles indicate the poorer performance and progress in educational attainment and economic status. Thereby, we infer that the poorer SDI states might shed light on the unequal distribution of social and health resources for mental disorders. In agreement with a few studies [51, 52], unequal economic development and health services in poorer regions might account for the deteriorating burden of diseases and explain the more prominent annualized rates of change in regions with lower SDIs. The finding that rural residents in some non-Greater Bay cities during 2010–2020 carried a higher disease burden than their urban counterparts may further account for the pronounced overall change of severe mental burden in these less developed regions. Though the Chinese government is pursuing a rural industry re-vitalization strategy to promote economic development in rural areas and to close the urban–rural gap in the healthcare systems [53], previous publications [54, 55] found that the level of urban–rural coordination development in the non-Greater Bay Areas of Guangdong remained relatively low while there being much room to promote the performance of public health services in rural areas. Nevertheless, our study would highly appeal to the proper redistribution of limited resources among various regions, such that balanced allocation of necessary resources for the urban and rural areas can be coordinated; and also appeal to the more active involvement of political, economic, social, and health professional stakeholders at the local communities, especially in the relatively less developed regions and rural areas. [18, 56, 57]

Population growth and aging may have pivotal roles in the dynamic trends of DALY. As hinted by several GBD studies [7, 58], these factors would fundamentally alter the distribution of disease burden associated with mental disorders. In Guangdong, though the total population has been increasing over the last few decades, most prefecture cities also rapidly entered the aging societies [59]. Our study shows that the population growth and aging explained 39.96% and 39.87%, respectively, of the increase of severe mental burden in the Greater Bay Area. For the non-Greater Bay Areas and especially the rural areas, the contribution of population and aging seems even greater, with a proportion exceeding 60%. To explain the aging effects from the spatial distribution of patients, we found that the numbers of the elderly in the rural areas were higher than those in the urban areas (shown in Additional file 1: Table S3). Since the Greater Bay Area is well recognized as an economically-developed region [17, 18], we infer that it may attract more migrant workers while the more elderly people and those with mental health disorders are more likely to be left behind in the rural communities in the non-Greater Bay Areas. It may in part be held accountable for the higher population aging effect among the non-Greater Bay Areas, much similar to previous findings on the societal aging in rural China [60]. These findings constantly remind the authorities and relevant stakeholders of the importance of considering regional population structure and aging when planning for future health needs. Specifically, we anticipate the rising trends for overall DALYs in Guangdong to persist into the future, where the DALY burden for the Greater Bay Area remains the heaviest. The projected rising burden shall warrant urgent prioritization of mental health programs focused on population health, prevention strategies, and continuous medical support in the basic and specialized public health services programs tailored for patients with severe mental disorders. However, we noted that the estimates on the scenario decomposition by population growth and by changing age structure were much alike. The similarities may in part reflect the crucial role of the aging effect in exacerbating the mental health burden, while also suggesting the less prominent role of population growth due to birth and death. Given the more pressing issues of entering aging societies in China [59] and the rising future burden, it is high time to plan for mental public health services with a healthy aging agenda and to promote health at the later stage of life for patients with severe mental health disorders.

In comparison with the GBD study [2], our estimated numbers of DALYs for patients with severe mental disorders took up nearly half of the GBD estimates (43.4%), the latter of which reported DALY estimates ascribed to both severe and non-severe mental disorders. These findings not only confirm the importance of severe mental disorders but also suggest that schizophrenia may be one of the leading mental disorders and that mental burden is primarily due to the non-fatal burden of YLD [2, 28, 29, 32]. In contrast to the GBD 2017 [2], we observed slightly higher estimates for the age-standardized DALY rates of schizophrenia in Guangdong, and also found overlapping uncertainty intervals between our estimates and those reported by the GBD 2017 (307.33 person-years [95%UI: 231.98, 376.55] per 100,000). This is probably due to our comprehensive evaluation of the disease patterns and comorbidities presented by the real-world GDMHS database (shown in Additional file 1: Table S1) and it put out a crucial signal for drawing the attention of relevant stakeholders to care for the coexistent conditions and multi-system diseases in severe mental patients. The overlapping uncertainty intervals nonetheless reflect the consistency and robustness of these age-standardized results. Compared with other provinces and municipalities at a similar level of development (such as Beijing, Shanghai, and Tianjin), Guangdong has slightly higher age-standardized DALY rates of schizophrenia, albeit their overlapping uncertainty intervals. The similarity in the scales of these uncertainty intervals may add credibility to the findings of the present study.

The findings in our study strengthen the previous research in several ways. Firstly, a key strength of this study is the very large number of follow-up data collected over the last decade. The follow-up data is a population-based database and offers a great amount of validated information on the clinical as well as epidemiological features of patients with severe mental disorders [18]. With its extensive coverage of over 4900 mental health-related institutes, DALYs estimated from the database would reflect the urgency of the burden of severe mental disorders in a more thorough manner. Secondly, to our knowledge, this is the first and latest regional burden of disease study detailing the DALY burden for sub-regions of Guangdong. Next, the study enriched the burden of disease study by combining the GBD comorbidity assessment method with locally observed data. We found that the difference between comorbidity-adjusted DALYs and non-adjusted DALYs was almost as twice much as the non-adjusted burden estimates. This nonetheless highlights the importance of consideration of local disease patterns and comorbidities [61] when estimating the DALY burden.

However, some limitations should be noted in our study. Firstly, YLL could not be estimated directly from the database, since the underlying causes of death were primarily due to diseases other than mental disorders (Table 1). Secondly, we did not conduct a complete risk analysis, since it is beyond the scope of the current paper. However, we have utilized the novel SDI indicator [40] to interpret the changing patterns in DALYs. Annualized rates of change in DALYs categorized by SDI quintiles were similar to those reported in GBD. Finally, we could only estimate DALYs based on country-level disability weight from the GBD. Since no regional disability weights were publicly available, we argue future research in evaluating the regional disability weights may be needed. Notwithstanding, the proposed comprehensive evaluation framework and modeling schemes developed as part of the research can yet be applied to the other regional burden of disease study throughout different areas of the world.

## Conclusions

This large-scale real-world follow-up study confirms the necessity of continuous support for prioritizing mental health services among patients with severe mental disorders. The study highlights the urgent need to redistribute health resources and prioritize mental health programs focused on population health and prevention strategies in places with low SDI states. With a drastic increase in population size, a transition towards aging societies, and an oversize population with severe mental disorders, the overall mental burden may become an enormous challenge for the authorities as well as relevant stakeholders. Our regional burden of disease study can be used by policy-makers and relevant stakeholders to plan for and improve the mental health strategies and services able to address the current as well as the future burden of mental disorders.

## Supplementary Information

**Additional file 1**. **Section 1**. Methods in details. **Section 2. Table S1**. Comorbidity list recorded by the GDMHS with disability weights extracted from the Global Burden of Disease (GBD) studies. **Table S2**. Ratios of years of life lost (YLL) to years lived with disability (YLD) for mental disorders, extracted from the Global Burden of Disease (GBD) studies, for 2010-2020. **Table S3**. Descriptive statistics of the collected study records (N = 520731), by sex and residence. **Table S4**. Average disability-adjusted life-years (DALYs) for patients with severe mental disorders in Guangdong, China, during 2010-2020, by residence and prefectural city. **Table S5**. Average years of life lost (YLLs) and years lived with disability (YLDs) for severe mental disorders in Guangdong province during 2010 ~ 2020, by sex and prefectural city. **Table S6**. Average years of life lost (YLLs) and years lived with disability (YLDs) for severe mental disorders in Guangdong province during 2010 ~ 2020, by residence and prefectural city. **Table S7**. Change in absolute DALY numbers and age-standardized rates (per 100000) for severe mental disorders in Guangdong province, China, between 2010 and 2020, by economic region, for females. **Table S8**. Change in absolute DALY numbers and age-standardized rates (per 100000) for severe mental disorders in Guangdong province, China, between 2010 and 2020, by economic region, for males. **Table S9**. Change in absolute DALY numbers and age-standardized rates (per 100000) for severe mental disorders in Guangdong province, China, between 2010 and 2020, by economic region, for the urban residents. **Table S10**. Change in absolute DALY numbers and age-standardized rates (per 100000) for severe mental disorders in Guangdong province, China, between 2010 and 2020, by economic region, for the rural residents. **Table S11**. Socio-demographic Index (SDI) for each prefecture and economic region in Guangdong province, China, between 2010 and 2020. **Table S12**. Evaluation metrics for different generalized additive model (GAM) based age smoothing functions, implemented in the epidemiological adjustment for prevalence estimates. **Table S13**. A range of ω used in the weighted median annualized rate of change formula, and the corresponding RMSE metrics for each city. **Figure S1**. Location of each local community health service center (CHSC) and psychiatric hospital within the Guangdong Mental Health center network medical System (GDMHS), in Guangdong, China, 2020. **Figure S2**. The estimated age-specific duration for mental disorders in Guangdong province, by sex, and by city. **Figure S3**. The average number of disorder-specific DALYs (person-years) for each economic region in Guangdong province, China, during 2010 ~ 2020, by age. **Figure S4**. Weighted median annualized rates of change (A) and change of logarithm of values (B) in disabilityadjusted life-years (DALYs) for patients with schizophrenia in Guangdong province, China, from 2010 to 2020, by SDI group. **Figure S5**. Weighted median annualized rates of change (A) and change of logarithm of values (B) in disability-adjusted life-years (DALYs) for patients with severe mental retardation in Guangdong province, China, from 2010 to 2020, by SDI group.

## Data Availability

The data that support the findings of this study are available from the corresponding authors, [HL and XL], upon reasonable request.

## Abbreviations

GBD: Global Burden of Disease
GATHER statement: Guidelines for Accurate and Transparent Health Estimates Reporting statement
GDMHS: Guangdong Mental Health System
YLL: Year of life lost
YLD: Year lived with disability
DALY: Disability-adjusted life-year
SDI: Socio-demographic Index
UI: Uncertainty interval
ICD-10: The 10th revision of the International Classification of Diseases
