# Supplementary material for: Regional years of life lost, years lived with disability, and disability-adjusted life-years for severe mental disorders in Guangdong Province, China: a real-world longitudinal study
Source: Glob Health Res Policy. 2022 Jun 20;7:17. doi: 10.1186/s41256-022-00253-3 (PMC9208127; doi:10.1186/s41256-022-00253-3)
Supplement: Supplementary file 1 — Additional file 1. Section 1. Methods in details. Section 2. Table S1. Comorbidity list recorded by the GDMHS with disability weights extracted from the Global Burden of Disease (GBD) studies. Table S2. Ratios of years of life lost (YLL) to years lived with disability (YLD) for mental disorders, extracted from the Global Burden of Disease (GBD) studies, for 2010-2020. Table S3. Descriptive statistics of the collected study records (N = 520731), by sex and residence. Table S4. Average disability-adjusted life-years (DALYs) for patients with severe mental disorders in Guangdong, China, during 2010-2020, by residence and prefectural city. Table S5. Average years of life lost (YLLs) and years lived with disability (YLDs) for severe mental disorders in Guangdong province during 2010 ~ 2020, by sex and prefectural city. Table S6. Average years of life lost (YLLs) and years lived with disability (YLDs) for severe mental disorders in Guangdong province during 2010 ~ 2020, by residence and prefectural city. Table S7. Change in absolute DALY numbers and age-standardized rates (per 100000) for severe mental disorders in Guangdong province, China, between 2010 and 2020, by economic region, for females. Table S8. Change in absolute DALY numbers and age-standardized rates (per 100000) for severe mental disorders in Guangdong province, China, between 2010 and 2020, by economic region, for males. Table S9. Change in absolute DALY numbers and age-standardized rates (per 100000) for severe mental disorders in Guangdong province, China, between 2010 and 2020, by economic region, for the urban residents. Table S10. Change in absolute DALY numbers and age-standardized rates (per 100000) for severe mental disorders in Guangdong province, China, between 2010 and 2020, by economic region, for the rural residents. Table S11. Socio-demographic Index (SDI) for each prefecture and economic region in Guangdong province, China, between 2010 and 2020. Table S12. Evaluation metrics for different g [file 41256_2022_253_MOESM1_ESM.docx]

Regional years of life lost, years lived with disability, and disability-adjusted life-years for severe mental disorders in Guangdong Province, China: A real-world longitudinal study

Wenyan Tan^a,1^, Lichang Chen^b,1^, Yuqin Zhang^b^, Junyan Xi^b^, Yuantao Hao^c**^, Fujun Jia^a,**^, Brian J. Hall^d^, Jing Gu^b,e^, Shibin Wang^a^, Haicheng Lin^a,*^, and Xiao Lin^b,e,1,*^

^a^Guangdong Mental Health Center, Guangdong Provincial People’s Hospital, Guangdong Academy of Medical Sciences, Guangzhou, China.

^b^Department of Medical Statistics & Center for Health Information Research & Guangdong Key Laboratory of Medicine, School of Public Health, Sun Yat-sen University, Guangzhou, 510080, Guangdong, China.

^c^Peking University Center for Public Health and Epidemic Preparedness & Response, Peking University, 100191, Beijing, China.

^d^Global Public Health, New York University (Shanghai), Shanghai, 200122, China.

^e^Sun Yat-sen Global Health Institute, Sun Yat-sen University, Guangzhou, 510080, Guangdong, China.

^1^ Wenyan Tan, Lichang Chen, and Xiao Lin contributed equally to this work.

**^*^Address correspondence to** Doctor Xiao Lin, Sun Yat-sen University, 74 Zhongshan 2^nd^ Rd, Guangzhou, 510080, Guangdong, China. E-mail: linx87@mail.sysu.edu.cn. Doctor Haicheng Lin, Guangdong Mental Health Center, Guangdong, China. E-mail: haichenglin@189.cn.

^**^ Co-senior authors.

**Competing Financial Interests**: The authors declare they have no actual or potential competing financial interests.

**Contents**

Methods in details……………………………………………………………….… 3

Table S1.…………………………………………………………………………… 8

Table S2.…………………………………………………………………………… 9

Table S3.…………………………………………………………………………… 10

Table S4.…………………………………………………………………………… 14

Table S5.…………………………………………………………………………… 16

Table S6.……………………………………………………………….…………… 17

Table S7.…………………………………………………………………………… 18

Table S8.…………………………………………………………………………… 19

Table S9.…………………………………………………………………………… 20

Table S10.………………………………………………………………….....…… 21

Table S11.…………………………………………………………………..……… 22

Table S12.…………………………………………………………………….…… 24

Table S13.…………………………………………………………………….…… 28

Figure S1.……………………………………………………………………..…… 30

Figure S2.………………………………………………………………………..… 32

Figure S3.………………………………………………………………………..… 34

Figure S4.………………………………………………………………………..… 35

Figure S5.………………………………………………………………………..… 37

References…………………………………………………………………….…… 39

Methods in details

Data collection

We collected valid registered medical records from Guangdong Mental Health System (GDMHS), covering January 1, 2010, to December 24, 2020. From the surveillance-based system, de-identified medical information was extracted on mental health disorders diagnosed by ICD-10 (the 10th revision of the International Classification of Diseases ^1^) codes (F00-F99). Each record within GDMHS was validated and confirmed by the Guangdong Health Commission ^2,3^. Our target individuals consisted of 520,731 persons that were clinically diagnosed with severe mental disorders including schizophrenia, schizoaffective disorder, delusional disorder, bipolar disorder, psychotic disorder due to epilepsy, and mental retardation with mental disorders ^4,5^. These disorders were chosen according to the Chinese definition of severe mental disorders ^6,7^. More details concerning information from GDMHS can be found elsewhere ^8^.

The dataset included: a) deidentified individual-level demographic information such as the residential address, age, biological gender, education level, marital and employment status; b) a follow-up medical record of diagnosis for mental disorders and relevant comorbidities (as shown in S1 Table); c) family and genetic history of diagnosed mental disorders; and d) survival status (coded in ICD-10).

Epidemiological adjustment

To generate internally consistent epidemiological estimates, the age-sex-city-specific prevalence estimates were further adjusted by an epidemiological system dynamics model similar to the DisMod-MR model designed specifically for the Global burden of Disease (GBD) purposes ^9,10^. Consistent with the GBD study, we chose a two-compartment framework as the basic system dynamics model to account for the process of disease in the targeted subjects [equation (1-2)]. The two-compartment model comprises susceptible (*S*) and condition (*C*) compartments. *S* refers to the population susceptible to mental disorders and *C* contains the population with mental disorders. Under the framework of the system dynamics model, individuals can move from *S* to *C* with incidence hazard $h_{i}$, and from *C* to *S* with remission hazard $h_{r}$. Individuals from *S* can also flow out of the system with baseline mortality hazard $h_{m}$ ^11^, whereas individuals from *C* can flow out of the system with with-condition mortality hazard $h_{m}+h_{f}$. $h_{f}$ refers to the cause-specific mortality hazard, quantifying the excessive mortality for individuals with mental disorders. Finally, the adjusted age-sex-city-specific prevalence of mental disorders was calculated using the equation $p\left( a,t \right)=C/(S+C)$ [equation (3)].

$\frac{d}{d\tau}S\left( a+\tau,t+\tau\right)=-\left( h_{i}+h_{m} \right)S+h_{r}C$ (1)

$\frac{d}{d\tau}C\left( a+\tau,t+\tau\right)=h_{i}S-(h_{r}+h_{m}+h_{f})C$ (2)

To solve the differential equations, we considered the parameters of the model as functions of age ($a$) and time ($t$). For each age group, we used fourth-order Runge–Kutta algorithm ^12^ to approximate the value of model parameters over different years. Model parameters were determined by minimizing dispersion and dissipation errors over a given range of frequencies such as prevalence, remission rates, and mortality rates in the compartments. Then, to properly interpolate variability and reflect changing patterns of the model parameters between different age groups, we proposed a generalized additive model (GAM) based age smoothing function to generate consistent estimates across age and time. Under the GBD framework, DisMod-MR used a Bayesian penalized spline model for age pattern smoothing and needed the cumbersome work of choosing the number of spline knots as well as Bayesian priors for different ages ^10^. Compared with the GBD method, our GAM-based age smoothing function can: a) utilize hierarchical variability from different sex groups and cities; and b) estimate the smoothness parameters for the spline functions as fixed effects using the restricted maximum likelihood approach, a computationally efficient data-driven smoothness selection algorithm in generalized additive modeling ^13,14^. Modeling assumptions, sources of data for compartment hazards, smoothing functions, as well as other relevant information on the analysis methodology are briefly summarized in Supplement Table S12 and Supplement Figure 2.

Comorbidity adjustment

In line with the GBD study, we applied comorbidity adjustment to the disability weight of mental disorders in the calculation of years lived with disability (YLDs) ^9,15^. Under the analysis framework, we performed comorbidity simulations separately for age-sex-city-specific estimates to account for the comorbidities amongst individuals. We synthesized a population of 20000 individuals ($n$) and exposed the synthetic population to the probability of having all the different comorbidities recorded in the real-world database. Specifically, a total of 11 comorbidities ($J=11$) were included in the follow-up records (S1 Table). Yet, unlike the GBD method utilizing hypothetical probability^16^, prevalence rates of comorbidities were empirically calculated ($p_{empirical}$) based on real-world observation in our study. Next, we treated the empirical prevalence as the probability of having a comorbidity for each simulant in the synthetic population, and determined the comorbidity status for each simulant ($simu=1$ indicates having the comorbidity disease while $simu=0$ indicates the opposite) based on a draw from the binomial distribution . With simulated disease status and latest disability weight (DW) values for the comorbidities from GBD, we were able to calculate the comorbidity-adjusted DW ($como-DW_{l}$) [equation (4)]. Finally, with the comorbidity-adjusted DW, comorbidity-adjusted YLD rates attributed to mental disorders were calculated using [equation (5)]. $DW_{k}$ is the disability weight for each of the $k$ comorbidity that simulant $l$ has obtained. To estimate the number of comorbidity-adjusted YLDs, the appropriate age-sex-city-year-specific population was applied to the age-sex-city-year-specific YLD rates. We also reported 95% uncertainty intervals (UI) for the burden of disease estimates in the paper. Sources of uncertainty for the burden of disease estimation included the uncertainty of data corrections for comorbidity, uncertainty in coefficients from the epidemiological modeling and smoothing, and uncertainty of disability weights ^17^. To propagate uncertainty throughout the comorbidity-adjusted YLD estimates ^18^, we drew a total of 1000 values from the uncertainty distribution of disability weight for each comorbidity and conducted a bootstrap analysis with 1000 repetitions for the abovementioned methods. Based on the bootstrap samples, 95% UIs were estimated as the 2.5^th^ and 97.5^th^ values of the posterior distribution.

$como-DW_{l}=1-\Pi_{k=1}^{J+1}(1-DW_{k}*simu)$ (4)

$como-YLD rate_{k}=\Sigma_{l=1}^{n}(\frac{DW_{k}}{\Sigma_{k=1}^{k=J+1}DW_{k}}*como-DW_{l})/n$ (5)

Decomposition and projection of disability-adjusted life-years (DALYs) over time

To explore the underlying reasons for changes in disease burden over time, we implemented the time decomposition method proposed by Charlson ^19^ for DALY ascribed to mental health disorders. Briefly, we first computed counterfactual DALY numbers based on a population growth scenario and a population aging scenario. Under the first scenario, we estimated the expected number of DALYs for each region by first applying the region-specific total population in 2020 to the age-sex structure of the region-specific population at 2010 levels, and then multiplying the estimated population by the age-sex-specific DALY crude rates in 2020. Thus, the percentage change between DALY estimates and scenario expected values reflects the change in absolute numbers due to population growth. As for the second scenario, expected DALYs in 2020 for each region were calculated using 2010 age-sex-specific DALY crude rates and age-sex-region-specific population numbers for 2020. The percentage change for the second scenario represents the change in absolute numbers due to changing age structure.

As a final step, we projected DALYs ascribed to mental disorders for each region of Guangdong to 2030, based on the weighted median annualized rate of change ^20,21^. In detail, we first calculated the annual rate of change between 2010 and 2020 for each year in natural-log space. Next, the weighted median annualized rate of change was calculated using [equation (6)]. In the weighting function, the value of $\omega$ denotes the number of weights given to recent years compared with past years. Detailed methods concerning the rate of change were summarized in Supplement Table S13 and elsewhere ^20^.

${weight}_{year}=\frac{\left( year-2010 \right)^{\omega}}{\Sigma_{t=2011}^{T}\left( t-2010 \right)^{\omega}}$ (6)

Table S1. Comorbidity list recorded by the GDMHS with disability weights extracted from the Global Burden of Disease (GBD) studies^9^.

| **Condition/Cause^a^** | **Disability weights**  **(95% Confidence interval)** | **Similarity scores^b^** |
| --- | --- | --- |
| Mental disorders | 0.17 (0.11, 0.22) | 1.00 |
| Comorbidity |  |  |
| Malignant tumor (neoplasm) | 0.33 (0.24, 0.43) | 1.00 |
| Hepatitis | 0.19 (0.12, 0.25) | 1.00 |
| Hypertension | 0.25 (0.17, 0.33) | 0.98 |
| Coronary heart disease | 0.22 (0.15, 0.29) | 1.00 |
| Tuberculosis | 0.31 (0.22, 0.40) | 0.92 |
| Chronic obstructive pulmonary disease (COPD) | 0.24 (0.16, 0.31) | 1.00 |
| Stroke | 0.36 (0.26, 0.45) | 0.97 |
| Other notifiable infectious diseases | 0.12 (0.08, 0.17) | 1.00 |
| Diabetes | 0.28 (0.20, 0.36) | 1.00 |
| Occupational diseases | 0.36 (0.26, 0.46) | 0.99 |
| Other diseases or conditions | 0.23 (0.16, 0.30) | 1.00 |

^a^ A total of 12 conditions or causes were recorded in the follow-up archives collected via the Guangdong Mental Health center network medical System (GDMHS) ^8^, including records for mental disorders and comorbidities.

^b^ Disability weights were extracted from the GBD 2019 study by using matching records (including the ‘Sequela’ column and ‘Health state name’ column) similar to those in keywords of the 12 above conditions. To avoid subjective bias in extraction, the degree of match was determined by the Jaro-Winkler distance computed by the stringdist package in R (version 3.6) ^22,23^. Matching records of sequelae and health states whose similarity scores were above 0.9 were extracted ^24^, after which mean and confidence interval were computed. For subsequent bootstrap analysis of uncertainty (see section ‘2.5 Comorbidity adjustment’ in the main manuscript), a triangular distribution was specified for the uncertainty distribution of disability weight for each comorbidity^18,25,26^.

Table S2. Ratios of years of life lost (YLL) to years lived with disability (YLD) for mental disorders, extracted from the Global Burden of Disease (GBD) studies, for 2010-2020^9^.

| **Age_start** | **Age_end** | **YLL/YLD ratio** | |
| --- | --- | --- | --- |
|  |  | **Male** | **Female** |
| 0 | 4 | 0 | 0 |
| 5 | 9 | 0.000073 | 0.000072 |
| 10 | 14 | 0.000003 | 0.000256 |
| 15 | 19 | 0.000122 | 0.000319 |
| 20 | 24 | 0.000239 | 0.000333 |
| 25 | 29 | 0.000060 | 0.000303 |
| 30 | 34 | 0.000158 | 0.000174 |
| 35 | 39 | 0.000098 | 0.000178 |
| 40 | 44 | 0.000217 | 0.000133 |
| 45 | 49 | 0.000178 | 0.000129 |
| 50 | 54 | 0 | 0 |
| 55 | 59 | 0 | 0 |
| 60 | 64 | 0 | 0 |
| 65 | 69 | 0 | 0 |
| 70 | 74 | 0 | 0 |
| 75 | 79 | 0 | 0 |
| 80 | 84 | 0 | 0 |
| 85 | 99 | 0 | 0 |

Notes: Data on YLL and YLD for mental disorders can be extracted from the GBD Results Tool at <http://ghdx.healthdata.org/gbd-results-tool>. In the GBD study, YLLs were estimated for mental disorders such as schizophrenia and eating disorders ^27^, because only these disorders were considered by the ICD-10 as direct causes of death ^1^. Given the available GBD estimates on both YLLs and YLDs, Sex-specific YLL/YLD ratios were simply the number of sex-specific YLLs divided by the number of sex-specific YLDs ^9^.

Table S3. Descriptive statistics of the collected study records (N = 520731), by sex and residence.

| **Variables** | **Male**  **N (%)** | **Female**  **N (%)** | **Urban**  **N (%)** | **Rural**  **N (%)** |
| --- | --- | --- | --- | --- |
| **Age group (years)** |  |  |  |  |
| <7 | 2305 (0.8) | 1042 (0.5) | 1793 (1.1) | 1554 (0.4) |
| 7~12 | 7603 (2.6) | 3541 (1.5) | 4533 (2.8) | 6611 (1.9) |
| 13~17 | 12586 (4.3) | 8237 (3.6) | 6869 (4.2) | 13954 (3.9) |
| 18~44 | 1  72644 (59.3) | 112739 (49.1) | 87245 (53.0) | 198138 (55.7) |
| 45~64 | 82917 (28.5) | 84207 (36.7) | 53351 (32.4) | 113773 (32.0) |
| ≥65 | 13228 (4.5) | 19682 (8.6) | 10952 (6.6) | 21958 (6.2) |
| **Sex** |  |  |  |  |
| Female | 0 (0.0) | 229448 (100.0) | 73130 (44.4) | 156318 (43.9) |
| Male | 291283 (100.0) | 0 (0.0) | 91613 (55.6) | 199670 (56.1) |
| **Residence** |  |  |  |  |
| Rural | 199670 (68.5) | 156318 (68.1) | 0 (0.0) | 355988 (100.0) |
| Urban | 91613 (31.5) | 73130 (31.9) | 164743 (100.0) | 0 (0.0) |
| **Region** |  |  |  |  |
| Pearl River Delta (Greater Bay Areas) | 125060 (53.4) | 109000 (46.6) | 105136 (44.9) | 128924 (55.1) |
| North Guangdong | 48511 (55.6) | 38784 (44.4) | 15624 (17.9) | 71671 (82.1) |
| West Guangdong | 66162 (57.3) | 49241 (42.7) | 20908 (18.1) | 94495 (81.9) |
| East Guangdong | 51550 (61.4) | 32423 (38.6) | 23075 (27.5) | 60898 (72.5) |
| **Educational level** |  |  |  |  |
| Illiteracy | 59559 (20.4) | 55973 (24.4) | 28557 (17.3) | 86975 (24.4) |
| Elementary school | 92380 (31.7) | 81615 (35.6) | 44908 (27.3) | 129087 (36.3) |
| Junior high school | 87152 (29.9) | 53967 (23.5) | 44328 (26.9) | 96791 (27.2) |
| Senior high school or specialized secondary school | 28045 (9.6) | 18201 (7.9) | 22785 (13.8) | 23461 (6.6) |
| Tertiary school or university or higher | 9267 (3.2) | 8100 (3.5) | 10833 (6.6) | 6534 (1.8) |
| Others | 14880 (5.1) | 11592 (5.1) | 13332 (8.1) | 13140 (3.7) |
| **Marital status** |  |  |  |  |
| Unmarried | 181468 (62.3) | 59577 (26.0) | 77739 (47.2) | 163306 (45.9) |
| Married | 88253 (30.3) | 143916 (62.7) | 68274 (41.4) | 163895 (46.0) |
| Divorced | 11230 (3.9) | 9089 (4.0) | 8310 (5.0) | 12009 (3.4) |
| Widowed | 2555 (0.9) | 12597 (5.5) | 4387 (2.7) | 10765 (3.0) |
| Unspecified | 7777 (2.7) | 4266 (1.9) | 6032 (3.7) | 6011 (1.7) |
| **Employment status** |  |  |  |  |
| Unemployed | 287221 (98.6) | 226388 (98.7) | 160345 (97.3) | 353264 (99.2) |
| Employed ^a^ | 4054 (1.4) | 3052 (1.3) | 4391 (2.7) | 2715 (0.8) |
| Unspecified | 8 (0.0) | 8 (0.0) | 7 (0.0) | 9 (0.0) |
| **Economical level** |  |  |  |  |
| Poverty ^b^ | 144957 (49.8) | 101862 (44.4) | 67954 (41.2) | 178865 (50.2) |
| Non-poverty | 146326 (50.2) | 127586 (55.6) | 96789 (58.8) | 177123 (49.8) |
| **Mental disorders by ICD ^c^ groups** |  |  |  |  |
| F00-F09: Organic, including symptomatic, mental disorders | 17830 (6.1) | 11554 (5.0) | 8801 (5.3) | 20583 (5.8) |
| F10-F19: Mental and behavioral disorders due to psychoactive substance use | 3315 (1.1) | 170 (0.1) | 1179 (0.7) | 2306 (0.6) |
| F20-F29: Schizophrenia, schizotypal and delusional disorders | 181433 (62.3) | 151933 (66.2) | 98253 (59.6) | 235113 (66.0) |
| F30-F39: Mood [affective] disorders | 22149 (7.6) | 24532 (10.7) | 22688 (13.8) | 23993 (6.7) |
| F40-F48: Neurotic, stress-related, and somatoform disorders | 316 (0.1) | 436 (0.2) | 307 (0.2) | 445 (0.1) |
| F50-F59: Behavioral syndromes associated with physiological disturbances and physical factors | 56 (0.0) | 56 (0.0) | 38 (0.0) | 74 (0.0) |
| F60-F69: Disorders of adult personality and behavior | 99 (0.0) | 26 (0.0) | 50 (0.0) | 75 (0.0) |
| F70-F79: Mental retardation (Intellectual disabilities) ^d^ | 65504 (22.5) | 40455 (17.6) | 33046 (20.1) | 72913 (20.5) |
| F80-F89: Disorders of psychological development | 216 (0.1) | 35 (0.0) | 145 (0.1) | 106 (0.0) |
| F90-F98, F99: Behavioral and emotional disorders with onset usually occurring in childhood and adolescence, and unspecified mental disorder | 365 (0.1) | 251 (0.1) | 236 (0.1) | 380 (0.1) |
| **Family history** | |  | | --- | |  |  |  |
| No | 262217 (90.0) | 206583 (90.0) | 148163 (89.9) | 320637 (90.1) |
| Yes | 17420 (6.0) | 13986 (6.1) | 8702 (5.3) | 22704 (6.4) |
| Unspecified | 11646 (4.0) | 8879 (3.9) | 7878 (4.8) | 12647 (3.6) |
| **Screening for genetic history** |  |  |  |  |
| No | 286918 (98.5) | 225615 (98.3) | 162860 (98.9) | 349673 (98.2) |
| Yes | 4291 (1.5) | 3776 (1.6) | 1802 (1.1) | 6265 (1.8) |
| Unspecified | 74 (0.0) | 57 (0.0) | 81 (0.0) | 50 (0.0) |
| **Deceased** ^e^ | 5406 (1.9) | 3892 (1.7) | 2922 (1.8) | 6376 (1.8) |
| Cerebrovascular diseases (I60-I69) | 700 (12.9) | 503 (12.9) | 360 (12.3) | 843 (13.2) |
| Cardiovascular diseases (I20-I25, I26-I51) | 589 (10.9) | 464 (11.9) | 330 (11.3) | 723 (11.3) |
| Diseases of the respiratory system (J00-J98) | 375 (6.9) | 224 (5.8) | 214 (7.3) | 385 (6.0) |
| Neoplasms (C00-D48) | 298 (5.5) | 201 (5.2) | 165 (5.6) | 334 (5.2) |
| Diseases of the digestive system (K00-K92) | 171 (3.2) | 74 (1.9) | 78 (2.7) | 167 (2.6) |
| Other causes (excluding psychotic disorders) | 3273 (60.5) | 2426 (62.3) | 1775 (60.7) | 3924 (61.5) |

^a^ Employed workers include professional technology personnel; clerks and relevant personnel; manufacturing and related personnel; agriculture, forestry, husbandry and fishery production, and auxiliary personnel, *etc..*

^b^ Poverty is defined as the residents and households living under the urban and rural subsistence allowance.

^c^ ICD: World Health Organization International Classification of Diseases, 10th revision.

^d^ According to the work specification for management and treatment of severe mental disorders (2018 edition), severe mental patients with mental retardation are referred to as patients having mental retardation with mental disorders in the Chinese context.

^e^  Mental patients in the study were provided with follow-up services by local community health centers, from which death information regarding the causes of death was extracted. Causes of death were also coded according to ICD-10.

Table S4. Average disability-adjusted life-years (DALYs) for patients with severe mental disorders in Guangdong, China, during 2010-2020, by residence and prefectural city.

| **Prefectural city** | **DALY (non-adjusted)** | | **DALY (comorbidity-adjusted)** | | **Age-standardized^a^ DALY rate (per 100000, non-adjusted)** | | **Age-standardized DALY rate (per 100000, comorbidity-adjusted)** | |
| --- | --- | --- | --- | --- | --- | --- | --- | --- |
|  | **Urban (95% UI^b^)** | **Rural (95% UI)** | **Urban (95% UI)** | **Rural (95% UI)** | **Urban (95% UI)** | **Rural (95% UI)** | **Urban (95% UI)** | **Rural (95% UI)** |
| **Guangzhou** | 12146 (8259, 16717) | 12915 (8783, 17661) | 88299 (56870, 143965) | 94791 (61407, 154255) | 93.39 (63.50, 128.52) | 99.58 (67.74, 136.12) | 664.98 (421.96, 1090.45) | 715.16 (456.55, 1170.21) |
| **Shenzhen** | 6477 (4348, 8972) | 7161 (4848, 9823) | 81200 (52421, 132675) | 69400 (44220, 114056) | 57.73 (38.81, 79.88) | 62.51 (42.31, 85.76) | 741.34 (471.88, 1218.16) | 610.70 (383.07, 1009.94) |
| **Foshan** | 9222 (6419, 12261) | 9340 (6505, 12425) | 61950 (41210, 99303) | 60852 (40474, 97456) | 130.42 (90.79, 173.38) | 131.37 (91.47, 174.79) | 847.35 (555.39, 1365.86) | 827.62 (542.36, 1333.37) |
| **Dongguan** | 8985 (5850, 12421) | 6820 (4464, 9890) | 32563 (21488, 50809) | 22681 (14656, 36664) | 117.50 (76.57, 162.35) | 88.60 (57.97, 128.57) | 409.45 (267.64, 640.56) | 284.31 (182.10, 461.20) |
| **Zhanjiang** | 4627 (2990, 6621) | 22008 (15359, 29112) | 9258 (5761, 15430) | 38387 (25554, 61425) | 69.20 (44.67, 99.05) | 329.26 (229.75, 435.53) | 133.31 (81.89, 223.39) | 550.80 (361.12, 886.32) |
| **Zhuhai** | 1677 (1153, 2334) | 1306 (882, 1799) | 17939 (12162, 30587) | 14096 (8932, 23206) | 100.99 (69.57, 140.56) | 78.10 (52.72, 107.69) | 1077.39 (723.58, 1855.01) | 845.28 (526.70, 1400.81) |
| **Qingyuan** | 1825 (1053, 2793) | 9915 (6909, 13155) | 4523 (2526, 7898) | 23665 (15719, 37889) | 51.71 (29.82, 79.16) | 280.67 (195.57, 372.44) | 122.32 (67.63, 214.68) | 638.38 (418.10, 1027.38) |
| **Meizhou** | 2477 (1153, 4179) | 12582 (8693, 16854) | 5102 (2575, 9434) | 21410 (13985, 34506) | 62.03 (28.88, 104.65) | 315.01 (217.53, 421.91) | 122.65 (61.41, 227.87) | 511.73 (329.45, 829.33) |
| **Jiangmen** | 5265 (3665, 7008) | 11546 (8054, 15211) | 6525 (4317, 10499) | 14269 (9497, 22863) | 125.78 (87.57, 167.40) | 275.79 (192.41, 363.38) | 148.87 (97.05, 240.85) | 325.36 (213.41, 524.12) |
| **Huizhou** | 4230 (2898, 5756) | 8905 (6212, 11800) | 5697 (3689, 9269) | 12035 (7970, 19354) | 96.26 (65.95, 130.96) | 202.64 (141.38, 268.48) | 123.87 (79.07, 202.62) | 261.64 (170.74, 422.99) |

Table S4. Average disability-adjusted life-years (DALYs) for patients with severe mental disorders in Guangdong, China, during 2010-2020, by residence and prefectural city (continued).

| **Prefectural city** | **DALY (non-adjusted)** | | **DALY (comorbidity-adjusted)** | | **Age-standardized^a^ DALY rate (per 100000, non-adjusted)** | | **Age-standardized DALY rate (per 100000, comorbidity-adjusted)** | |
| --- | --- | --- | --- | --- | --- | --- | --- | --- |
|  | **Urban (95% UI^b^)** | **Rural (95% UI)** | **Urban (95% UI)** | **Rural (95% UI)** | **Urban (95% UI)** | **Rural (95% UI)** | **Urban (95% UI)** | **Rural (95% UI)** |
| **Jieyang** | 2088 (1243, 3715) | 11118 (7571, 14572) | 2976 (1617, 5696) | 13516 (8716, 21606) | 37.53 (22.32, 66.73) | 199.77 (136.06, 261.82) | 51.15 (27.50, 98.33) | 231.70 (147.37, 372.30) |
| **Shaoguan** | 3374 (2219, 4764) | 8849 (6159, 11776) | 4150 (2589, 6922) | 10981 (7261, 17666) | 124.09 (81.67, 175.22) | 325.41 (226.54, 433.05) | 146.01 (89.91, 244.73) | 386.63 (251.86, 625.66) |
| **Zhongshan** | 2886 (2011, 3823) | 1853 (1285, 2480) | 8389 (5565, 13486) | 4839 (3173, 7821) | 96.50 (67.21, 127.79) | 61.31 (42.53, 82.08) | 269.81 (176.36, 436.11) | 154.13 (99.63, 250.53) |
| **Maoming** | 4179 (2115, 6734) | 19348 (13421, 25872) | 2318 (1176, 4199) | 9457 (6148, 15421) | 73.59 (37.39, 118.46) | 339.01 (235.20, 453.29) | 39.39 (19.87, 71.60) | 158.90 (101.82, 260.58) |
| **Heyuan** | 1694 (1067, 2471) | 7466 (5208, 9889) | 2294 (1388, 3873) | 9247 (6114, 14874) | 59.96 (37.79, 87.43) | 264.31 (184.35, 350.12) | 77.98 (46.56, 132.55) | 313.34 (204.26, 507.04) |
| **Shantou** | 2606 (1762, 3589) | 6050 (4214, 8038) | 4337 (2784, 7113) | 6992 (4560, 11348) | 50.81 (34.37, 69.96) | 117.60 (81.91, 156.23) | 81.66 (51.64, 134.75) | 130.42 (83.82, 212.85) |
| **Zhaoqing** | 2852 (1863, 4035) | 11706 (8162, 15472) | 2323 (1454, 3866) | 8619 (5700, 13869) | 75.88 (49.60, 107.33) | 310.59 (216.52, 410.52) | 59.44 (36.69, 99.40) | 219.34 (142.88, 354.79) |
| **Yangjiang** | 2543 (1558, 3757) | 6804 (4702, 9152) | 2195 (1294, 3754) | 5536 (3612, 8963) | 109.40 (67.03, 161.70) | 292.39 (202.05, 393.37) | 90.48 (52.72, 155.56) | 227.62 (146.46, 370.59) |
| **Yunfu** | 1444 (948, 2033) | 8525 (5942, 11234) | 919 (572, 1534) | 5262 (3482, 8453) | 63.18 (41.51, 88.92) | 371.77 (259.22, 489.89) | 38.52 (23.68, 64.53) | 219.73 (143.34, 354.82) |
| **Chaozhou** | 1266 (791, 1842) | 3058 (2112, 4113) | 1847 (1116, 3123) | 3575 (2301, 5850) | 51.75 (32.34, 75.32) | 125.15 (86.42, 168.36) | 71.83 (42.79, 122.12) | 138.93 (88.20, 228.78) |
| **Shanwei** | 1870 (1166, 2749) | 3768 (2579, 5134) | 1411 (838, 2420) | 2450 (1546, 4058) | 67.99 (42.39, 99.93) | 136.99 (93.72, 186.62) | 49.04 (28.74, 84.56) | 84.92 (52.84, 141.50) |

^a^ Estimated value for age-standardized rates was calculated based on the Chinese population reported by the GBD 2019 ^28^.

^b^ UI: uncertainty interval.

Table S5. Average years of life lost (YLLs) and years lived with disability (YLDs) for severe mental disorders in Guangdong province during 2010 ~ 2020, by sex and prefectural city.

| **Prefectural city** | **YLL (non-adjusted)** | | **YLL (comorbidity-adjusted)** | | **YLD (non-adjusted)** | | **YLD**  **(comorbidity-adjusted)** | |
| --- | --- | --- | --- | --- | --- | --- | --- | --- |
|  | **Male** | **Female** | **Male** | **Female** | **Male** | **Female** | **Male** | **Female** |
| **Guangzhou** | 0 (0, 1) | 0 (0, 0) | 8 (5, 14) | 11 (7, 20) | 14008 (9778, 18317) | 11052 (7715, 14451) | 95467 (64045, 152315) | 87602 (58754, 139739) |
| **Shenzhen** | 0 (0, 0) | 0 (0, 0) | 7 (4, 12) | 9 (6, 17) | 7607 (5310, 9946) | 6031 (4210, 7885) | 78568 (52633, 125342) | 72015 (48228, 114846) |
| **Foshan** | 0 (0, 0) | 0 (0, 0) | 6 (3, 10) | 8 (4, 13) | 10357 (7230, 13543) | 8204 (5727, 10727) | 64035 (42908, 102059) | 58754 (39361, 93616) |
| **Dongguan** | 0 (0, 0) | 0 (0, 0) | 4 (2, 7) | 6 (3, 10) | 8822 (6158, 11534) | 6983 (4875, 9130) | 49291 (33030, 78543) | 45213 (30285, 72027) |
| **Zhanjiang** | 1 (0, 3) | 0 (0, 1) | 1 (0, 3) | 2 (0, 3) | 14854 (10368, 19422) | 11779 (8222, 15401) | 24853 (16621, 39694) | 22788 (15236, 36383) |
| **Zhuhai** | 0 (0, 0) | 0 (0, 0) | 0 (0, 1) | 0 (0, 1) | 1671 (1167, 2185) | 1325 (926, 1733) | 16163 (10853, 25773) | 14827 (9952, 23633) |
| **Qingyuan** | 0 (0, 0) | 0 (0, 0) | 0 (0, 1) | 1 (0, 1) | 6548 (4572, 8562) | 5192 (3624, 6787) | 14355 (9596, 22896) | 13197 (8819, 21039) |
| **Jiangmen** | 0 (0, 0) | 0 (0, 0) | 0 (0, 1) | 0 (0, 1) | 9385 (6551, 12271) | 7426 (5184, 9709) | 13985 (9339, 22387) | 12859 (8583, 20577) |
| **Meizhou** | 0 (0, 0) | 0 (0, 0) | 0 (0, 1) | 0 (0, 1) | 8403 (5866, 10987) | 6655 (4645, 8700) | 13256 (8861, 21205) | 12154 (8121, 19438) |
| **Jieyang** | 0 (0, 0) | 0 (0, 0) | 0 (0, 0) | 0 (0, 1) | 7364 (5141, 9629) | 5842 (4078, 7638) | 12561 (8356, 20112) | 11557 (7686, 18497) |
| **Huizhou** | 0 (0, 0) | 0 (0, 0) | 0 (0, 0) | 0 (0, 1) | 7333 (5119, 9587) | 5802 (4050, 7585) | 10737 (7149, 17192) | 9863 (6566, 15789) |
| **Yunfu** | 0 (0, 0) | 0 (0, 0) | 0 (0, 1) | 1 (1, 2) | 5561 (3882, 7271) | 4407 (3077, 5762) | 10139 (6773, 16184) | 9363 (6251, 14942) |
| **Shaoguan** | 0 (0, 0) | 0 (0, 0) | 0 (0, 0) | 0 (0, 0) | 6825 (4764, 8924) | 5398 (3768, 7057) | 9143 (6108, 14621) | 8396 (5606, 13424) |
| **Yangjiang** | 0 (0, 0) | 0 (0, 0) | 0 (0, 0) | 0 (0, 1) | 5214 (3640, 6818) | 4132 (2885, 5403) | 6810 (4536, 10890) | 6271 (4176, 10025) |
| **Zhongshan** | 0 (0, 0) | 0 (0, 0) | 0 (0, 0) | 0 (0, 0) | 2646 (1847, 3459) | 2094 (1461, 2737) | 6596 (4393, 10571) | 6048 (4026, 9689) |
| **Maoming** | 0 (0, 1) | 0 (0, 0) | 0 (0, 0) | 0 (0, 0) | 13130 (9165, 17169) | 10396 (7257, 13592) | 5964 (3935, 9616) | 5474 (3611, 8823) |
| **Heyuan** | 0 (0, 0) | 0 (0, 0) | 0 (0, 0) | 0 (0, 0) | 5110 (3568, 6682) | 4051 (2829, 5297) | 5760 (3836, 9226) | 5282 (3517, 8459) |
| **Shantou** | 0 (0, 0) | 0 (0, 0) | 0 (0, 0) | 0 (0, 0) | 4824 (3368, 6306) | 3832 (2676, 5010) | 5591 (3681, 9035) | 5124 (3372, 8277) |
| **Zhaoqing** | 0 (0, 0) | 0 (0, 0) | 0 (0, 0) | 0 (0, 0) | 8127 (5673, 10625) | 6431 (4489, 8408) | 5439 (3604, 8752) | 4988 (3304, 8023) |
| **Chaozhou** | 0 (0, 0) | 0 (0, 0) | 0 (0, 0) | 0 (0, 0) | 2413 (1684, 3154) | 1911 (1334, 2499) | 2687 (1767, 4346) | 2464 (1620, 3985) |
| **Shanwei** | 0 (0, 0) | 0 (0, 0) | 0 (0, 0) | 0 (0, 0) | 3142 (2195, 4110) | 2496 (1743, 3264) | 1892 (1233, 3078) | 1734 (1129, 2819) |

Table S6. Average years of life lost (YLLs) and years lived with disability (YLDs) for severe mental disorders in Guangdong province during 2010 ~ 2020, by residence and prefectural city.

| **Prefectural city** | **YLL (non-adjusted)** | | **YLL (comorbidity-adjusted)** | | **YLD (non-adjusted)** | | **YLD**  **(comorbidity-adjusted)** | |
| --- | --- | --- | --- | --- | --- | --- | --- | --- |
|  | **Urban** | **Rural** | **Urban** | **Rural** | **Urban** | **Rural** | **Urban** | **Rural** |
| **Guangzhou** | 0 (0, 1) | 0 (0, 1) | 9 (5, 17) | 10 (6, 18) | 12146 (8259, 16716) | 12914 (8783, 17660) | 88289 (56864, 143948) | 94781 (61401, 154237) |
| **Shenzhen** | 0 (0, 0) | 0 (0, 0) | 9 (5, 15) | 7 (4, 14) | 6477 (4348, 8972) | 7161 (4848, 9823) | 81191 (52415, 132660) | 69392 (44215, 114043) |
| **Foshan** | 0 (0, 0) | 0 (0, 0) | 7 (4, 12) | 7 (4, 12) | 9222 (6419, 12261) | 9340 (6505, 12425) | 61944 (41206, 99292) | 60845 (40470, 97445) |
| **Dongguan** | 0 (0, 0) | 0 (0, 0) | 6 (3, 10) | 4 (2, 8) | 8985 (5850, 12421) | 6820 (4464, 9890) | 56038 (35249, 91739) | 38466 (23560, 65336) |
| **Zhanjiang** | 0 (0, 1) | 2 (0, 3) | 0 (0, 1) | 2 (1, 5) | 4627 (2990, 6621) | 22006 (15358, 29109) | 9257 (5761, 15429) | 38384 (25553, 61420) |
| **Zhuhai** | 0 (0, 0) | 0 (0, 0) | 0 (0, 1) | 0 (0, 1) | 1677 (1153, 2334) | 1306 (882, 1799) | 17358 (11399, 28727) | 13434 (8665, 21923) |
| **Qingyuan** | 0 (0, 0) | 0 (0, 0) | 0 (0, 0) | 1 (0, 2) | 1825 (1053, 2793) | 9915 (6909, 13155) | 4307 (2387, 7546) | 23245 (15439, 37222) |
| **Meizhou** | 0 (0, 0) | 0 (0, 0) | 0 (0, 0) | 0 (0, 1) | 2477 (1153, 4179) | 12582 (8693, 16853) | 4169 (1969, 7919) | 21241 (13931, 34163) |
| **Jiangmen** | 0 (0, 0) | 0 (0, 0) | 0 (0, 1) | 0 (0, 1) | 5265 (3665, 7008) | 11546 (8054, 15211) | 9598 (6362, 15415) | 17247 (11478, 27632) |
| **Huizhou** | 0 (0, 0) | 0 (0, 0) | 0 (0, 0) | 0 (0, 1) | 4230 (2898, 5756) | 8905 (6212, 11800) | 6867 (4450, 11172) | 13733 (9087, 22091) |
| **Jieyang** | 0 (0, 0) | 0 (0, 0) | 0 (0, 1) | 0 (0, 1) | 2088 (1243, 3715) | 11118 (7571, 14571) | 8711 (5340, 14979) | 15407 (9665, 25066) |
| **Shaoguan** | 0 (0, 0) | 0 (0, 0) | 0 (0, 0) | 0 (0, 0) | 3374 (2219, 4764) | 8849 (6159, 11776) | 4633 (2860, 7770) | 12907 (8533, 20768) |
| **Zhongshan** | 0 (0, 0) | 0 (0, 0) | 0 (0, 0) | 0 (0, 0) | 2886 (2011, 3823) | 1853 (1285, 2480) | 8028 (5324, 12915) | 4616 (3027, 7466) |
| **Maoming** | 0 (0, 0) | 1 (0, 1) | 0 (0, 0) | 0 (0, 0) | 4179 (2115, 6734) | 19347 (13421, 25870) | 2092 (1033, 3832) | 9346 (6084, 15230) |
| **Heyuan** | 0 (0, 0) | 0 (0, 0) | 0 (0, 0) | 0 (0, 0) | 1694 (1067, 2471) | 7466 (5208, 9889) | 2053 (1232, 3482) | 8989 (5946, 14459) |
| **Shantou** | 0 (0, 0) | 0 (0, 0) | 0 (0, 0) | 0 (0, 0) | 2606 (1762, 3589) | 6050 (4214, 8038) | 3881 (2484, 6377) | 6833 (4463, 11083) |
| **Zhaoqing** | 0 (0, 0) | 0 (0, 0) | 0 (0, 0) | 0 (0, 0) | 2852 (1863, 4035) | 11706 (8162, 15472) | 2160 (1345, 3605) | 8267 (5458, 13322) |
| **Yangjiang** | 0 (0, 0) | 0 (0, 0) | 0 (0, 1) | 0 (0, 0) | 2543 (1558, 3757) | 6804 (4702, 9152) | 6572 (4165, 10792) | 6508 (4032, 10804) |
| **Yunfu** | 0 (0, 0) | 0 (0, 0) | 0 (0, 1) | 1 (0, 2) | 1444 (948, 2033) | 8525 (5942, 11234) | 6115 (4022, 9883) | 13387 (8913, 21406) |
| **Chaozhou** | 0 (0, 0) | 0 (0, 0) | 0 (0, 0) | 0 (0, 0) | 1266 (791, 1842) | 3058 (2112, 4113) | 1617 (966, 2756) | 3534 (2285, 5770) |
| **Shanwei** | 0 (0, 0) | 0 (0, 0) | 0 (0, 0) | 0 (0, 0) | 1870 (1166, 2749) | 3768 (2579, 5134) | 1280 (756, 2203) | 2346 (1483, 3882) |

Table S7. Change in absolute DALY^a^ numbers and age-standardized^b^ rates (per 100000) for severe mental disorders in Guangdong province, China, between 2010 and 2020, by economic region, for females.

| **Region** | **2010 DALYs (95% UI^c^)** | | **2020 DALYs (95% UI)** | | **Change in absolute numbers due to population growth (%)** | **Change in absolute numbers due to changing age structure (%)** | **Overall change 2010–2020 (%)^*^** |
| --- | --- | --- | --- | --- | --- | --- | --- |
|  | **Numbers** | **Age-standardized rates**  **(per 100000)** | **Numbers** | **Age-standardized rates**  **(per 100000)** |  |  |  |
| **Pearl River Delta^d^** | 173666 (116468, 277290) | 674.66 (444.11, 1086.01) | 331155 (224276, 521028) | 1116.43 (746.75, 1763.77) | 40.06 | 39.96 | 65.48 |
| **North Guangdong^e^** | 11851 (7897, 18911) | 189.03 (123.68, 304.17) | 53232 (35803, 84429) | 802.40 (532.47, 1278.01) | 76.81 | 76.78 | 324.49 |
| **West Guangdong^f^** | 13043 (8648, 20837) | 161.68 (105.17, 260.61) | 40894 (27380, 65165) | 470.18 (310.85, 752.44) | 65.96 | 65.92 | 190.82 |
| **East Guangdong^g^** | 5967 (3950, 9579) | 77.47 (50.34, 125.44) | 22737 (15096, 36342) | 282.90 (185.52, 453.94) | 73.09 | 73.06 | 265.16 |

^a^ DALY: disability-adjusted life-years, for which comorbidity-adjusted estimates were reported;

^b^ Estimated values for age-standardized rates were calculated based on the Chinese population reported by the GBD 2019 ^28^;

^c^ UI: uncertainty interval;

^d^ Pearl River Delta region (also known as the Greater Bay Area as mentioned in the main manuscript): including Guangzhou, Foshan, Zhaoqing, Shenzhen, Dongguan, Huizhou, Zhuhai, Zhongshan, Jiangmen;

^e^ North Guangdong region: referring to Shaoguan, Qingyuan, Meizhou, Heyuan;

^f^ West Guangdong region: referring to Zhanjiang, Yangjiang, Maoming, and Yunfu;

^g^ East Guangdong region: referring to Chaozhou, Shantou, Jieyang, and Shanwei;

^*^ DALY rates are age-standardized and sex-standardized so change in rates reflects changes in factors related to prevalence rates in addition to population growth and age structure.

Table S8. Change in absolute DALY^a^ numbers and age-standardized^b^ rates (per 100000) for severe mental disorders in Guangdong province, China, between 2010 and 2020, by economic region, for males.

| **Region** | **2010 DALYs (95% UI^c^)** | | **2020 DALYs (95% UI)** | | **Change in absolute numbers due to population growth (%)** | **Change in absolute numbers due to changing age structure (%)** | **Overall change 2010–2020 (%)^*^** |
| --- | --- | --- | --- | --- | --- | --- | --- |
|  | **Numbers** | **Age-standardized rates**  **(per 100000)** | **Numbers** | **Age-standardized rates**  **(per 100000)** |  |  |  |
| **Pearl River Delta^d^** | 188195 (126264, 300375) | 670.92 (442.46, 1080.26) | 359472 (243691, 565180) | 1107.64 (741.99, 1750.73) | 39.88 | 39.79 | 65.09 |
| **North Guangdong^e^** | 13301 (8888, 21183) | 194.61 (127.90, 312.45) | 57630 (38765, 91430) | 794.00 (527.22, 1266.51) | 75.86 | 75.83 | 308.00 |
| **West Guangdong^f^** | 14190 (9418, 22651) | 161.42 (105.26, 260.07) | 44283 (29647, 70578) | 465.29 (307.81, 745.69) | 65.64 | 65.60 | 188.25 |
| **East Guangdong^g^** | 6712 (4461, 10743) | 79.93 (52.22, 128.98) | 24610 (16354, 39338) | 279.88 (183.79, 449.68) | 71.91 | 71.88 | 250.14 |

^a^ DALY: disability-adjusted life-years, for which comorbidity-adjusted estimates were reported;

^b^ Estimated values for age-standardized rates were calculated based on the Chinese population reported by the GBD 2019 ^28^;

^c^ UI: uncertainty interval;

^d^ Pearl River Delta region(also known as the Greater Bay Area as mentioned in the main manuscript): including Guangzhou, Foshan, Zhaoqing, Shenzhen, Dongguan, Huizhou, Zhuhai, Zhongshan, Jiangmen;

^e^ North Guangdong region: referring to Shaoguan, Qingyuan, Meizhou, Heyuan;

^f^ West Guangdong region: referring to Zhanjiang, Yangjiang, Maoming, and Yunfu;

^g^ East Guangdong region: referring to Chaozhou, Shantou, Jieyang, and Shanwei;

^*^ DALY rates are age-standardized and sex-standardized so change in rates reflects changes in factors related to prevalence rates in addition to population growth and age structure.

Table S9. Change in absolute DALY^a^ numbers and age-standardized^b^ rates (per 100000) for severe mental disorders in Guangdong province, China, between 2010 and 2020, by economic region, for the urban residents.

| **Region** | **2010 DALYs (95% UI^c^)** | | **2020 DALYs (95% UI)** | | **Change in absolute numbers due to population growth (%)** | **Change in absolute numbers due to changing age structure (%)** | **Overall change 2010–2020 (%)^*^** |
| --- | --- | --- | --- | --- | --- | --- | --- |
|  | **Numbers** | **Age-standardized rates**  **(per 100000)** | **Numbers** | **Age-standardized rates**  **(per 100000)** |  |  |  |
| **Pearl River Delta^d^** | 176969 (112228, 293509) | 329.07 (205.08, 550.45) | 310480 (197261, 508974) | 499.60 (313.57, 822.05) | 34.67 | 34.56 | 51.82 |
| **North Guangdong^e^** | 15398 (9957, 25137) | 117.42 (74.64, 193.32) | 19218 (9567, 35243) | 138.21 (68.33, 254.80) | 16.29 | 16.12 | 17.70 |
| **West Guangdong^f^** | 14162 (9237, 22949) | 84.03 (53.88, 137.30) | 13891 (7343, 24850) | 76.17 (40.01, 137.15) | -9.06 | -9.17 | -9.36 |
| **East Guangdong^g^** | 9236 (6021, 15006) | 57.36 (36.74, 93.96) | 9326 (5295, 16789) | 55.36 (31.17, 100.10) | -1.77 | -1.86 | -3.49 |

^a^ DALY: disability-adjusted life-years, for which comorbidity-adjusted estimates were reported;

^b^ Estimated values for age-standardized rates were calculated based on the Chinese population reported by the GBD 2019 ^28^;

^c^ UI: uncertainty interval;

^d^ Pearl River Delta region (also known as the Greater Bay Area as mentioned in the main manuscript): including Guangzhou, Foshan, Zhaoqing, Shenzhen, Dongguan, Huizhou, Zhuhai, Zhongshan, Jiangmen;

^e^ North Guangdong region: referring to Shaoguan, Qingyuan, Meizhou, Heyuan;

^f^ West Guangdong region: referring to Zhanjiang, Yangjiang, Maoming, and Yunfu;

^g^ East Guangdong region: referring to Chaozhou, Shantou, Jieyang, and Shanwei;

^*^ DALY rates are age-standardized and sex-standardized so change in rates reflects changes in factors related to prevalence rates in addition to population growth and age structure.

Table S10. Change in absolute DALY^a^ numbers and age-standardized^b^ rates (per 100000) for severe mental disorders in Guangdong province, China, between 2010 and 2020, by economic region, for the rural residents.

| **Region** | **2010 DALYs (95% UI^c^)** | | **2020 DALYs (95% UI)** | | **Change in absolute numbers due to population growth (%)** | **Change in absolute numbers due to changing age structure (%)** | **Overall change 2010–2020 (%)^*^** |
| --- | --- | --- | --- | --- | --- | --- | --- |
|  | **Numbers** | **Age-standardized rates**  **(per 100000)** | **Numbers** | **Age-standardized rates**  **(per 100000)** |  |  |  |
| **Pearl River Delta^d^** | 190265 (119752, 314716) | 353.58 (218.73, 589.37) | 378079 (248500, 606585) | 608.84 (396.09, 981.23) | 42.32 | 42.23 | 72.19 |
| **North Guangdong^e^** | 9691 (5780, 16304) | 74.02 (43.56, 125.51) | 91428 (60695, 146658) | 657.95 (431.57, 1060.15) | 88.95 | 88.94 | 788.83 |
| **West Guangdong^f^** | 13048 (8483, 21249) | 77.39 (49.50, 127.07) | 71166 (47180, 113953) | 390.71 (255.80, 628.46) | 80.38 | 80.35 | 404.88 |
| **East Guangdong^g^** | 3409 (1977, 5921) | 21.16 (12.08, 37.05) | 37846 (24891, 61011) | 224.87 (146.14, 364.21) | 90.74 | 90.73 | 962.61 |

^a^ DALY: disability-adjusted life-years, for which comorbidity-adjusted estimates were reported;

^b^ Estimated values for age-standardized rates were calculated based on the Chinese population reported by the GBD 2019 ^28^;

^c^ UI: uncertainty interval;

^d^ Pearl River Delta region (also known as the Greater Bay Area as mentioned in the main manuscript): including Guangzhou, Foshan, Zhaoqing, Shenzhen, Dongguan, Huizhou, Zhuhai, Zhongshan, Jiangmen;

^e^ North Guangdong region: referring to Shaoguan, Qingyuan, Meizhou, Heyuan;

^f^ West Guangdong region: referring to Zhanjiang, Yangjiang, Maoming, and Yunfu;

^g^ East Guangdong region: referring to Chaozhou, Shantou, Jieyang, and Shanwei;

^*^ DALY rates are age-standardized and sex-standardized so change in rates reflects changes in factors related to prevalence rates in addition to population growth and age structure.

Table S11. Socio-demographic Index (SDI) for each prefecture and economic region in Guangdong province, China, between 2010 and 2020.

| **City/Region** | **Socio-demographic Index** |
| --- | --- |
| **Shenzhen** | 0.8497 |
| **Dongguan** | 0.7076 |
| **Zhongshan** | 0.5739 |
| **Zhuhai** | 0.5661 |
| **Guangzhou** | 0.5608 |
| **Foshan** | 0.5218 |
| **Huizhou** | 0.4049 |
| **Jiangmen** | 0.3024 |
| **Yangjiang** | 0.2358 |
| **Qingyuan** | 0.2269 |
| **Shaoguan** | 0.2218 |
| **Shantou** | 0.1786 |
| **Zhaoqing** | 0.1641 |
| **Yunfu** | 0.1407 |
| **Shanwei** | 0.1283 |
| **Heyuan** | 0.1139 |
| **Zhanjiang** | 0.1101 |
| **Chaozhou** | 0.1088 |
| **Jieyang** | 0.0920 |
| **Maoming** | 0.0762 |
| **Meizhou** | 0.0000 |
| **Pearl River Delta (median)** | 0.5608 |
| **North Guangdong (median)** | 0.1678 |
| **West Guangdong (median)** | 0.1254 |
| **East Guangdong (median)** | 0.1186 |

Notes: In line with the previous GBD study ^21,28,29^, we implemented the Socio-demographic Index (SDI) in the manuscript, to reflect the different levels of development and progression for each city and region. SDI is a summary measure of the overall development that was originally introduced in the GBD 2015 study ^21^. Similar to the Human Development Index (which is also a summary measure of average achievement in key dimensions of human development including life expectancy, educational attainment, and GNI per capita ^30,31^), SDI is computed by taking the geometric mean of three rescaled components: total fertility rate, lag-distributed income per capita, and average educational attainment. We extracted data from the Guangdong Statistical Yearbook ^32^ to compute the three rescaled indices ^17^: a) for total fertility rate, annual number of children who would be born and the corresponding number of parturient women were extracted; b) for lag-distributed income per capita, gross domestic product (GDP) per capita was extracted, after which log and lag1 (lagged behind one year) values were computed; and c) for average educational attainment, in order to calculate mean years of education, the number of graduates by school type as well as the required number of years of schooling (primary schooling takes 6 years, junior secondary schooling takes 3 years, and senior secondary schooling takes 3 years) were extracted, and mean years of schooling were estimated as weighted arithmetic mean with the proportion of number of graduates by school type using the required number of years of schooling as weights, and then, mean years of schooling were multiplied by the proportion of populations aged 15 years or above to generate average years of education in the population over age 15 years. Next, these indices were scaled from 0 (lowest income status, fewest years of schooling, and highest fertility) to 1 (highest income, most years of schooling, and lowest fertility), using the min-max transformation ^17^. The composite SDI score is just the geometric mean of the abovementioned rescaled indices, for a given location year. Besides, following the GBD practices, we also defined the values of zero and one for each component of the index: a) zero represents the level below which we could not observe GDP per capita or educational attainment or above which we could not observe the total fertility rate; and b) one represents the maximum level of each index in which health outcomes cease to improve. As a composite, the prefecture city or region with an SDI of zero would have a theoretical minimum level of development relevant to these specific health outcomes, while a location with an SDI of one would have a theoretical maximum level of development relevant to the best health outcomes. The SDI states were then categorized into quintiles (namely, high, high-middle, middle, low-middle, and low SDI state groups) in the manuscript, in which cutoff values used to determine quintiles for analysis were empirically selected, based on the distribution of estimated SDI values across 21 prefecture cities in Guangdong. Finally, as reported in the manuscript, the association between SDI and the DALY indicator was estimated, based on the Pearson correlation proposed by the GBD study ^33^.

Table S12. Evaluation metrics for different generalized additive model (GAM) based age smoothing functions, implemented in the epidemiological adjustment for prevalence estimates.

| **Term** | **nobs^a^** | **df^b^** | **Df residual** | **logLik^c^** | **AIC^d^** | **BIC^e^** | **deviance** | **Deviance explained (%)** | **Consumption of time (seconds)** |
| --- | --- | --- | --- | --- | --- | --- | --- | --- | --- |
| age_mid^f^ + s(sex_id, bs = "re") + s(City, bs = "mrf", xt = list(nb = nb3)) | 798 | 21.5076 | 776.4924 | -6990.8861 | 14027.1811 | 14133.4857 | 1900246464.8027 | 48.8368 | 14.0 |
| age_mid + s(sex_id, bs = "re") + s(City_int, bs = "re") | 798 | 21.4803 | 776.5197 | -6990.7475 | 14027.0205 | 14133.5982 | 1899586421.3912 | 48.8545 | 13.7 |
| s(age_mid, bs = "tp") + s(sex_id, bs = "re") + s(City, bs = "mrf", xt = list(nb = nb3)) | 798 | 29.0517 | 768.9483 | -6885.7858 | 13833.6383 | 13978.9399 | 1460201169.7791 | 60.6848 | 13.2 |
| s(age_mid, bs = "tp") + s(sex_id, bs = "re") + s(City_int, bs = "re") | 798 | 29.0527 | 768.9473 | -6885.6534 | 13833.4937 | 13979.0768 | 1459716553.3485 | 60.6978 | 13.2 |
| s(age_mid, bs = "gp") + s(sex_id, bs = "re") + s(City, bs = "mrf", xt = list(nb = nb3)) | 798 | 29.0463 | 768.9537 | -6885.4895 | 13833.0078 | 13978.2208 | 1459117199.6339 | 60.7140 | 14.4 |
| s(age_mid, bs = "gp") + s(sex_id, bs = "re") + s(City_int, bs = "re") | 798 | 29.0149 | 768.9851 | -6885.3893 | 13832.8111 | 13978.0322 | 1458750971.1018 | 60.7238 | 13.8 |
| age_mid + s(sex_id, bs = "re") + s(City, bs = "mrf", xt = list(nb = nb3)) | 798 | 21.5159 | 776.4841 | -5988.7443 | 12022.9326 | 12129.3195 | 849.0988 | 64.8706 | 13.4 |
| age_mid + s(sex_id, bs = "re") + s(City_int, bs = "re") | 798 | 21.5720 | 776.4280 | -5988.2887 | 12022.0423 | 12128.4783 | 849.1236 | 64.9074 | 13.7 |
| age_mid + s(sex_id, bs = "re") + s(City, bs = "mrf", xt = list(nb = nb3)) | 798 | 22.9982 | 775.0018 | -273486.9725 | 547019.9447 | 547127.6325 | 540488.0322 | 66.8042 | 13.9 |
| age_mid + s(sex_id, bs = "re") + s(City_int, bs = "re") | 798 | 22.9983 | 775.0017 | -273486.9723 | 547019.9446 | 547127.6330 | 540488.0319 | 66.8042 | 12.9 |
| s(age_mid, bs = "tp") + s(sex_id, bs = "re") + s(City, bs = "mrf", xt = list(nb = nb3)) | 798 | 29.5745 | 768.4255 | -5729.7466 | 11521.4297 | 11666.4265 | 807.3153 | 80.9177 | 14.8 |
| s(age_mid, bs = "tp") + s(sex_id, bs = "re") + s(City_int, bs = "re") | 798 | 29.6049 | 768.3951 | -5729.4648 | 11520.8921 | 11665.9500 | 807.3428 | 80.9304 | 13.7 |
| s(age_mid, bs = "gp") + s(sex_id, bs = "re") + s(City, bs = "mrf", xt = list(nb = nb3)) | 798 | 30.1771 | 767.8229 | -5721.3659 | 11506.3924 | 11655.4255 | 805.5163 | 81.2985 | 13.8 |
| s(age_mid, bs = "gp") + s(sex_id, bs = "re") + s(City_int, bs = "re") | 798 | 30.2124 | 767.7876 | -5721.0226 | 11505.7442 | 11654.8670 | 805.5287 | 81.3136 | 14.2 |
| s(age_mid, bs = "tp") + s(sex_id, bs = "re") + s(City, bs = "mrf", xt = list(nb = nb3)) | 798 | 30.9977 | 767.0023 | -80194.1913 | 160450.3816 | 160595.5247 | 153902.4699 | 90.5476 | 13.2 |
| s(age_mid, bs = "tp") + s(sex_id, bs = "re") + s(City_int, bs = "re") | 798 | 30.9978 | 767.0022 | -80194.1912 | 160450.3816 | 160595.5252 | 153902.4696 | 90.5476 | 13.9 |
| s(age_mid, bs = "gp") + s(sex_id, bs = "re") + s(City, bs = "mrf", xt = list(nb = nb3)) | 798 | 32.9974 | 765.0026 | -69628.0594 | 139322.1189 | 139476.6285 | 132770.2062 | 91.8455 | 15.5 |
| s(age_mid, bs = "gp") + s(sex_id, bs = "re") + s(City_int, bs = "re") | 798 | 32.9976 | 765.0024 | -69628.0593 | 139322.1186 | 139476.6282 | 132770.2059 | 91.8455 | 13.1 |

Notes: For each age group, we used fourth-order Runge–Kutta algorithm^12^ to approximate the value of model parameters over different years. This time integration method has been optimized by Bogey and others^34,35^ as the classical algorithms for linear as well as nonlinear operators. Since differential equations from the epidemiological system dynamics model ^10,36^ were solved by fourth-order Runge–Kutta algorithm ^12^ for each age group separately, there is a need to interpolate variability and reflect changing patterns of the estimated model parameters between different age groups. Thus, in line with the Global Burden of Disease (GBD) methodology, we also used the age pattern smoothing function to generate consistent estimates across continuous age groups. Regarding the GBD DisMod-MR model, which was designed specifically for the GBD purposes ^9,10,15^, it used a Bayesian penalized spline model for age pattern smoothing and needed the cumbersome work in choosing the proper number of spline knots as well as Bayesian priors for different ages. Even though penalized splines with an appropriate smoothing parameter generally reduce the challenge of choosing a set of knots ^10^, in the implementation of DisMod-MR, an arbitrary choice of the amount of smoothness should be fed into the model (see the ‘process.py’ coding files from the GitHub webpage ^37^). Thus, the smoothness of DisMod-MR was still primarily based on expert knowledge and subjective judgment. To avoid the potentially biased process of choosing the number of spline knots and expert priors, unlike the GBD methodology, we instead proposed the GAM-based age smoothing function. The ultimate goal of our GAM-based age smoothing function is to properly interpolate variability and reflect changing patterns of the estimated model parameters between different age groups. And to do so, our proposed method needs to: a) utilize hierarchical variability from different sex groups and cities; and b) estimate the smoothness parameters for the spline functions using a more data-driven approach instead of the subjective prior choosing approach. Following previous research protocol and published modeling guidelines ^38,39^, we constructed the GAM-based age smoothing functions with a continuous age variable (age_mid), a binary sex variable (sex_id: 1=Male, 2=Female), and a categorical city variable (City_int or City, coded based on integers 1~21, for a total number of 21 prefecture cities). Poisson link function was chosen for the prevalence count estimates ^40^. A range of smoothing functions was implemented, including thin plate regression splines (tp), random effects (re) penalized smoothing, Markov random fields (mrf) smoothing, Gaussian process (gp) smooths. To evaluate the proper number of knots or the degree of smoothness, we used the restricted maximum likelihood approach, which is a widely-implemented data-driven smoothness selection algorithm in generalized additive modeling, and it is also computationally efficient ^13,14^. Based on the best two GAM models, which explained the largest proportion of deviance in the residuals, we averaged fitted estimates from both models to generate age-smoothed values for the prevalence counts. The above Table 3 presents the demonstration of the procedures for a single year of data inputted in the model. By doing so, we were able to further adjust the prevalence estimates for severe mental disorders. Based on the epidemiological adjusted and age-smoothed prevalence estimates, years lived with disability were calculated. Thereby, we consider our methods as one of the many strengths of the study. Our proposed method may be able to handle hierarchical information depicted in different sex groups and cities while being more computationally efficient.

^a^ nobs, number of observations used.

^b^ df, degrees of freedom used by the model.

^c^ logLik, the log-likelihood of the model.

^d^ AIC, Akaike's Information Criterion for the model.

^e^ BIC, Bayesian Information Criterion for the model.

^f^ age_mid, the midpoint of the 5-year age group. Consistent with the GBD methodology ^9,41^ and the World Health Organization standards^42^, we coded the original age variable as the 5-year age group, and thus we had a total number of 18 age groups starting from 0 to 85+ (0~,5~,10~,…,85+).

Table S13. A range of $\boldsymbol{\omega}$ used in the weighted median annualized rate of change formula, and the corresponding RMSE^a^ metrics for each city.

| $\boldsymbol{\omega}$ **values** | **0** | **0.2** | **0.4** | **0.6** | **0.8** | **1** | **1.2** | **1.4** | **1.6** | **1.8** | **2** |
| --- | --- | --- | --- | --- | --- | --- | --- | --- | --- | --- | --- |
| **Chaozhou** | 0.13003 | 0.12991 | 0.12981 | 0.12972 | 0.12965 | 0.12951 | 0.12939 | 0.12934 | 0.12971 | 0.12998 | 0.13056 |
| **Dongguan** | 0.03297 | 0.03301 | 0.03298 | 0.03294 | 0.03292 | 0.03293 | 0.03300 | 0.03302 | 0.03304 | 0.03308 | 0.03308 |
| **Foshan** | 0.08214 | 0.08122 | 0.08101 | 0.08068 | 0.08073 | 0.08070 | 0.08078 | 0.08106 | 0.08125 | 0.08151 | 0.08174 |
| **Guangzhou** | 0.04676 | 0.04680 | 0.04680 | 0.04679 | 0.04676 | 0.04682 | 0.04680 | 0.04677 | 0.04665 | 0.04663 | 0.04674 |
| **Heyuan** | 0.06546 | 0.06516 | 0.06519 | 0.06522 | 0.06532 | 0.06559 | 0.06569 | 0.06568 | 0.06601 | 0.06613 | 0.06650 |
| **Huizhou** | 0.10135 | 0.10028 | 0.10027 | 0.10028 | 0.10026 | 0.10056 | 0.10071 | 0.10126 | 0.10137 | 0.10160 | 0.10184 |
| **Jiangmen** | 0.10100 | 0.09980 | 0.09973 | 0.09957 | 0.09964 | 0.09981 | 0.10057 | 0.10095 | 0.10145 | 0.10193 | 0.10251 |
| **Jieyang** | 0.06921 | 0.06860 | 0.06869 | 0.06872 | 0.06885 | 0.06880 | 0.06926 | 0.06934 | 0.06953 | 0.06977 | 0.06997 |
| **Maoming** | 0.09243 | 0.09184 | 0.09154 | 0.09144 | 0.09120 | 0.09099 | 0.09113 | 0.09160 | 0.09258 | 0.09373 | 0.09401 |
| **Meizhou** | 0.04715 | 0.04707 | 0.04682 | 0.04678 | 0.04683 | 0.04674 | 0.04680 | 0.04696 | 0.04710 | 0.04724 | 0.04740 |
| **Qingyuan** | 0.36416 | 0.36363 | 0.36347 | 0.36356 | 0.36366 | 0.36367 | 0.36399 | 0.36417 | 0.36436 | 0.36455 | 0.36491 |
| **Shantou** | 0.10762 | 0.10788 | 0.10792 | 0.10803 | 0.10805 | 0.10809 | 0.10804 | 0.10788 | 0.10781 | 0.10763 | 0.10746 |
| **Shanwei** | 0.17660 | 0.17727 | 0.17717 | 0.17753 | 0.17733 | 0.17686 | 0.17667 | 0.17616 | 0.17576 | 0.17560 | 0.17541 |
| **Shaoguan** | 0.08466 | 0.08426 | 0.08422 | 0.08440 | 0.08451 | 0.08454 | 0.08457 | 0.08455 | 0.08452 | 0.08464 | 0.08473 |
| **Shenzhen** | 0.04039 | 0.04063 | 0.04062 | 0.04066 | 0.04064 | 0.04059 | 0.04061 | 0.04049 | 0.04041 | 0.04042 | 0.04046 |
| **Yangjiang** | 0.06275 | 0.06257 | 0.06269 | 0.06263 | 0.06261 | 0.06261 | 0.06284 | 0.06293 | 0.06312 | 0.06327 | 0.06333 |
| **Yunfu** | 0.09427 | 0.09284 | 0.09282 | 0.09284 | 0.09338 | 0.09395 | 0.09484 | 0.09477 | 0.09543 | 0.09607 | 0.09618 |
| **Zhanjiang** | 0.04573 | 0.04581 | 0.04583 | 0.04585 | 0.04581 | 0.04577 | 0.04576 | 0.04569 | 0.04563 | 0.04563 | 0.04566 |
| **Zhaoqing** | 0.08514 | 0.08477 | 0.08461 | 0.08446 | 0.08446 | 0.08453 | 0.08467 | 0.08469 | 0.08499 | 0.08535 | 0.08569 |
| **Zhongshan** | 0.09429 | 0.09407 | 0.09392 | 0.09424 | 0.09423 | 0.09436 | 0.09450 | 0.09475 | 0.09498 | 0.09509 | 0.09517 |
| **Zhuhai** | 0.04929 | 0.04864 | 0.04863 | 0.04873 | 0.04871 | 0.04891 | 0.04904 | 0.04910 | 0.04946 | 0.04990 | 0.05038 |

^a^ RMSE, root mean squared error.

Notes: In line with the Global Burden of Disease (GBD) methodology ^20,21^, we confined the values of $\omega$ in the range from 0 to 2 with an increment of 0.2. Using the [equation (6)] ${weight}_{year}=\frac{\left( year-2010 \right)^{\omega}}{\Sigma_{t=2011}^{T}\left( t-2010 \right)^{\omega}}$, and holding out the 2018-2020 dataset as test set, we used the 2010-2017 dataset as the training dataset and calculated the ${weight}_{year}$ values based on different values of $\omega$. Next, we multiplied ${weight}_{year}$ values by annualized rate of change for each individual year, and obtained the weighted annualized rate of change for each city and each year. And then, we computed the median values for each city during the 2010-2017 period and obtained the weighted median annualized rate of change for each city. Based on the weighted median annualized rate of change, we predicted the expected values for the 2018-2020 period, after which RMSEs were computed. Based on the minimized RMSE criterion ^20^, the best $\omega$ values were extracted and then implemented in the final projection of DALY estimates for the period 2021-2030. In the final projection, based on the best ω values, the 2030 projection values were acquired by applying the weighted median annualized rate of change to the 2020 values.

**
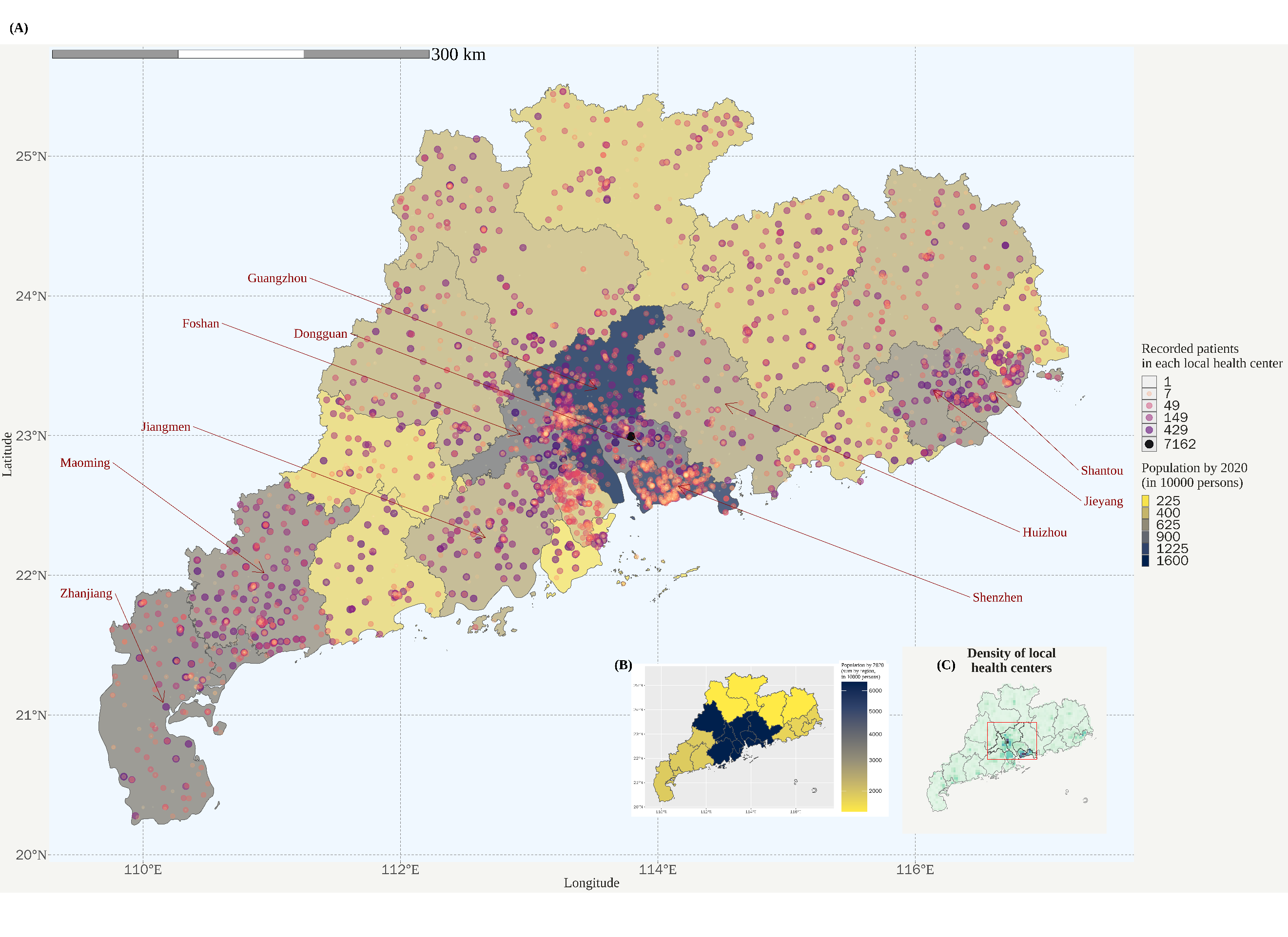
**

Figure S1. Location of each local community health service center (CHSC) and psychiatric hospital within the Guangdong Mental Health center network medical System (GDMHS), in Guangdong, China, 2020.

Notes: GDMHS had extensive coverage of 4980 local Community Health Service Centers (CHSC) and psychiatric hospitals (the location of each health facility was shown above). More details concerning the GDMHS can be found elsewhere ^8^. The location of each facility is shown on the main plot (A), with density map (C) in the corner. The darker blue color in the density map indicates the areas where health facilities are more densely-distributed. To indicate the number of records collected in the study, the quantile number of patients in each local health facility within the GDMHS was color-shaded. The bubble size on the map also reflects the number of records collected. Color filled within the boundary of each prefecture city reflects the range of population counts by 2020 (counted in 10000 persons). Prefecture cities with the top 10 population sizes are indicated by text labels on the map. Population aggregated by economic region was also plotted, as shown in panel (B).

**
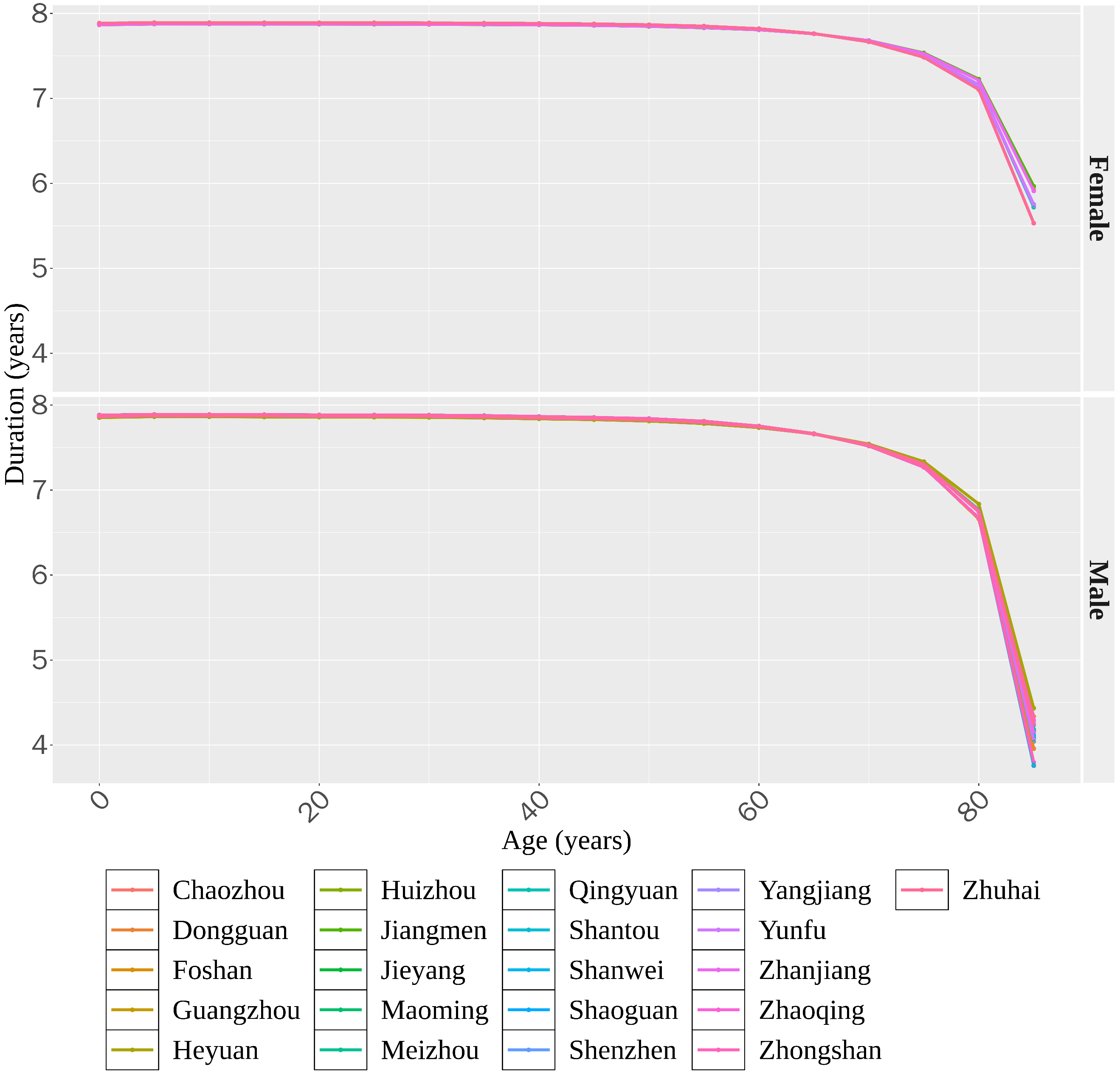
**

Figure S2. The estimated age-specific duration for mental disorders in Guangdong province, by sex, and by city.

Notes: Duration for mental disorders was generated by integrating the exponential negative summation of three hazard statuses (namely, remission hazard, cause-specific mortality hazard, and without-condition mortality hazard), over the time interval of 2010~2020, using the formula $duration_{t}=\int_{t=2010}^{2020} e^{^{-(h_{r}+h_{csmr}+h_{m})}}dt$. The hazard statuses were generated by the systematic compartmental model similar to the GBD (Global Burden of Disease) Dismod-MR-2.1 model ^10^. According to the literature review ^43,44^, the remission rate ($r$) was assumed at 33.2% for all ages combined in the current study. Cause-specific mortality rates ($csmr$) for mental disorders were extracted from the GBD 2019 study ^9^. Without-condition mortality hazard (also known as the baseline mortality hazard ^11^) was computed by $h_{m}=h_{m_{all}}-p\cdot h_{csmr}$, where all-cause mortality rate ($m_{all}$) was extracted from the GBD 2019. $p$ was the prevalence of mental disorders, estimated based on the collected study records.


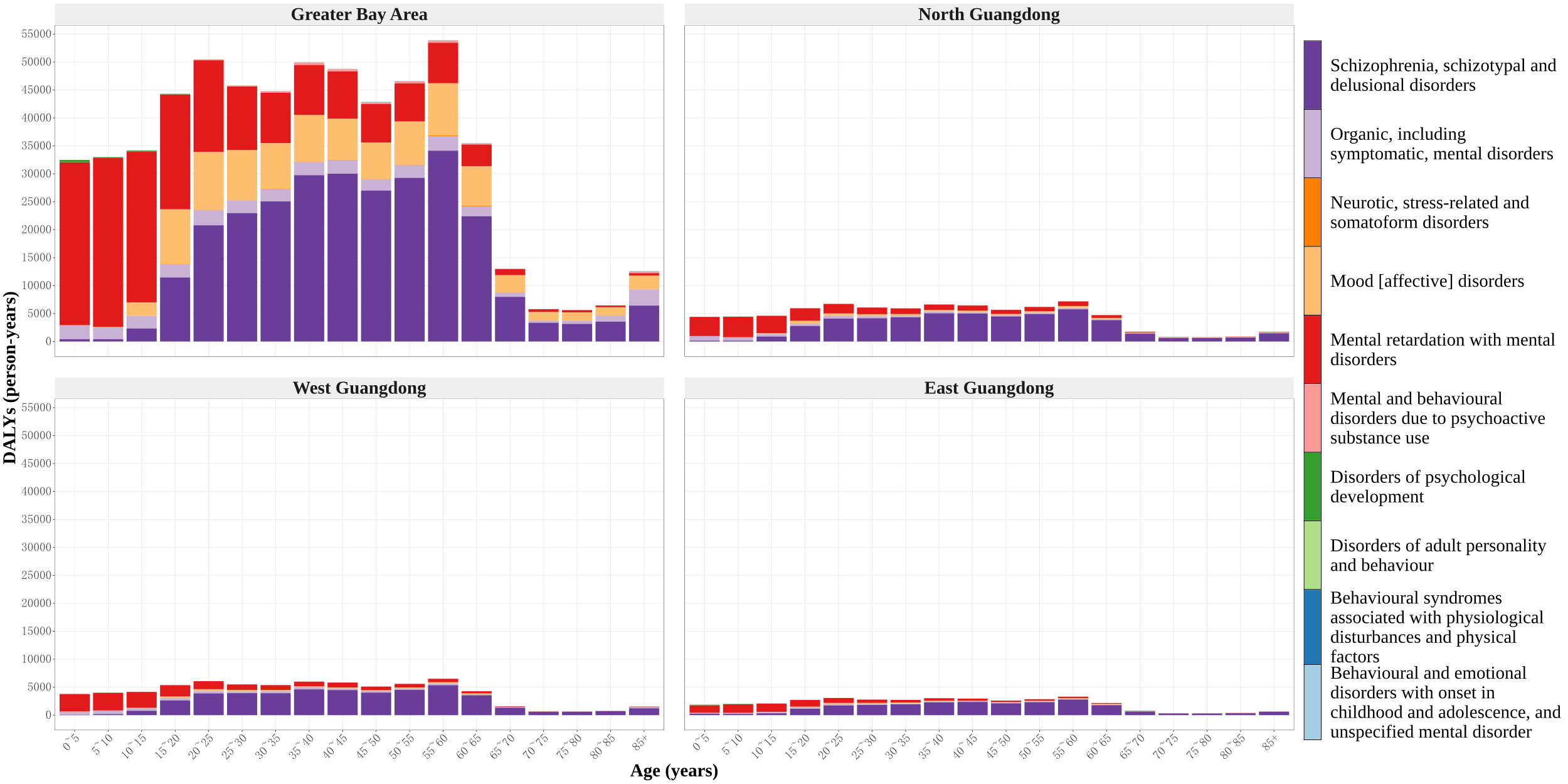


Figure S3. The average number of disorder-specific DALYs^a^ (person-years) for each economic region in Guangdong province, China, during 2010 ~ 2020, by age.

^a^ DALY: disability-adjusted life-years, for which comorbidity-adjusted estimates were reported.

Notes: DALYs for each region in the figure are ordered according to age-specific ranks. The Pearl River Delta region (also known as the Greater Bay Area as mentioned in the main manuscript) refers to Guangzhou, Foshan, Zhaoqing, Shenzhen, Dongguan, Huizhou, Zhuhai, Zhongshan, Jiangmen. The North Guangdong region refers to Shaoguan, Qingyuan, Meizhou, and Heyuan. The West Guangdong region refers to Zhanjiang, Yangjiang, Maoming and Yunfu. Finally, the East Guangdong region refers to Chaozhou, Shantou, Jieyang, and Shanwei. The figure shows ranks of DALY estimates among the four regions in Guangdong province, for a total of 18 age groups starting from 0 to 85+. Ranks are also color shaded to indicate rank intervals. Rank numbers whose DALY estimates fall into the zero-interval group were omitted. Finally, severe mental patients with mental retardation are referred to as patients having mental retardation with mental disorders under the Chinese definition of severe mental disorders ^4^.

**
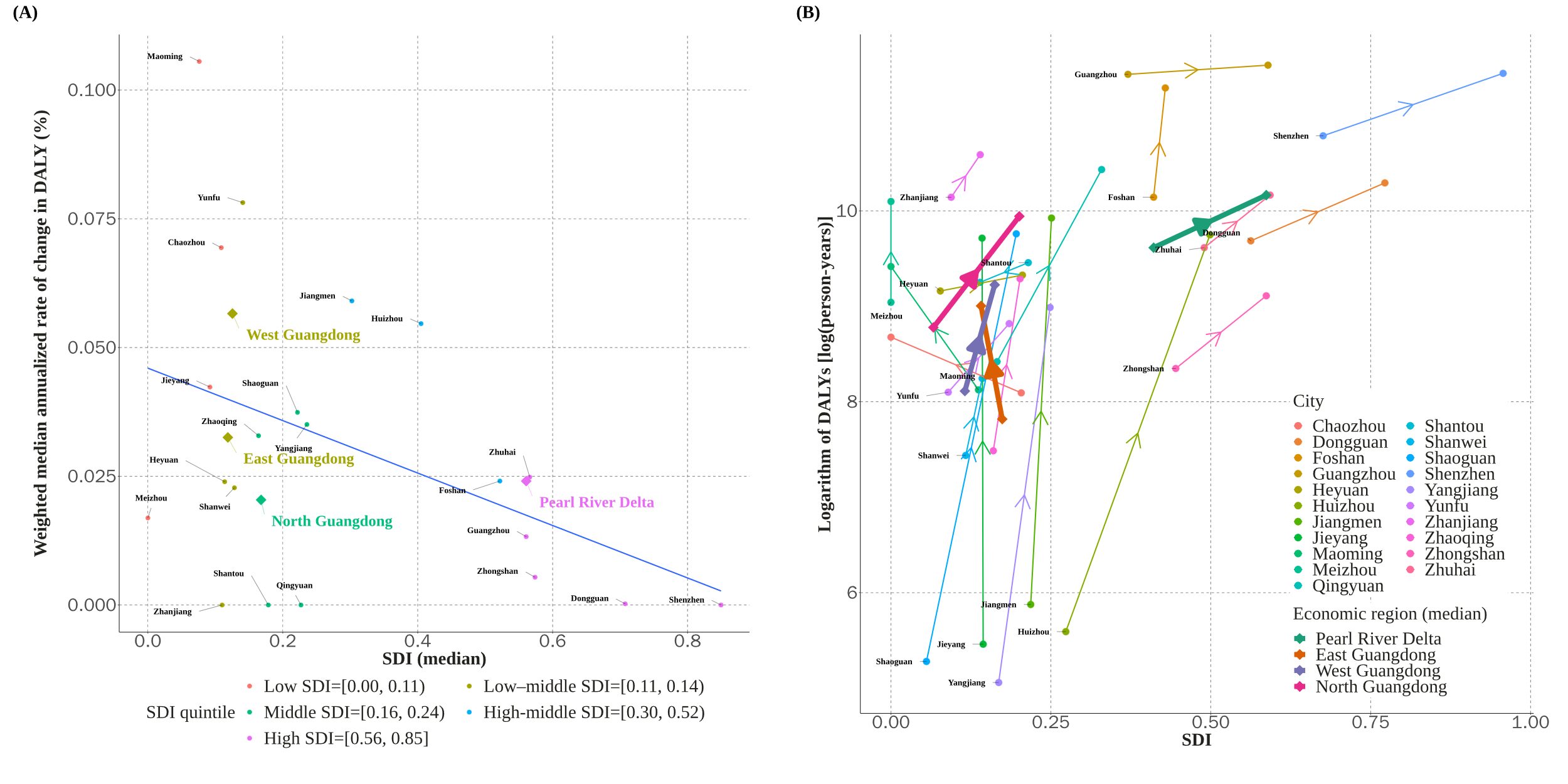
**

Figure S4. Weighted median annualized rates of change (A) and change of logarithm of values (B) in disability-adjusted life-years (DALYs) for patients with schizophrenia in Guangdong province, China, from 2010 to 2020, by SDI group.

Notes: Schizophrenia is coded as F20-F29 in the ICD-10 (the 10th revision of the International Classification of Diseases) codes. SDI = Socio-demographic Index. SDI groups were generated by calculating quintiles at the set of percentiles p={0, 0·2, 0·4, 0·6, 0·8, 1·0}, in which five categories of SDI were yielded (namely, low SDI < 0·11, Low-middle SDI <0·14, Middle SDI <0·24, high-middle SDI <0·52, and High SDI ≥0·56). In panel (A), the median of SDI during 2010-2020 was plotted, whereas the blue line reflects the linear relationship between estimated SDI and weighted median annualized rates of change in DALYs ascribed to severe mental disorders. A modest correlation between SDI values and annualized rates of change in DALYs was reported (r=-0·33, *p*=0·076). In panel (B), the logarithm of DALYs across 21 prefecture cities was further aggregated by the median for the four economic regions. Arrows indicate the direction of change in the logarithm of DALY, where comorbidity-adjusted values were reported.


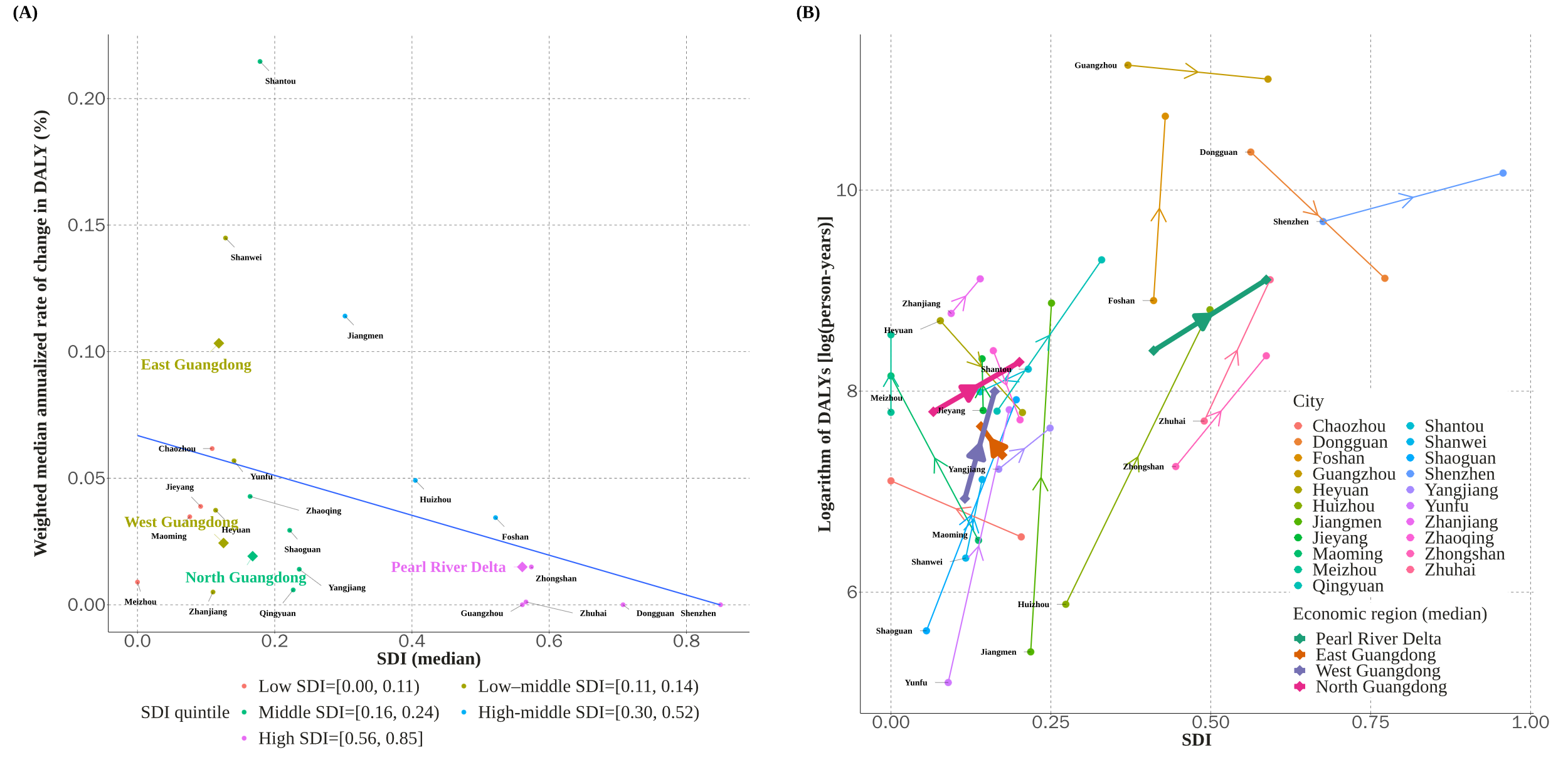


Figure S5. Weighted median annualized rates of change (A) and change of logarithm of values (B) in disability-adjusted life-years (DALYs) for patients with severe mental retardation in Guangdong province, China, from 2010 to 2020, by SDI group.

Notes: Mental retardation is coded as F70-F79 in the ICD-10 (the 10th revision of the International Classification of Diseases) codes. Under the Chinese definition, severe mental retardation refers to mental retardation with mental disorders ^4,6^. SDI = Socio-demographic Index. SDI groups were generated by calculating quintiles at the set of percentiles p={0, 0·2, 0·4, 0·6, 0·8, 1·0}, in which five categories of SDI were yielded (namely, low SDI < 0·11, Low-middle SDI <0·14, Middle SDI <0·24, high-middle SDI <0·52, and High SDI ≥0·56). In panel (A), the median of SDI during 2010-2020 was plotted, whereas the blue line reflects the linear relationship between estimated SDI and weighted median annualized rates of change in DALYs ascribed to severe mental disorders. A modest correlation between SDI values and annualized rates of change in DALYs was reported (r=-0·55, *p*=0·035). In panel (B), the logarithm of DALYs across 21 prefecture cities was further aggregated by the median for the four economic regions. Arrows indicate the direction of change in the logarithm of DALY, where comorbidity-adjusted values were reported.

References

1. WHO (World Health Organization). International Classification of Diseases, Tenth Revision (ICD-10); 2015.

2. Huang Y, Wang Y, Wang H, et al. Prevalence of mental disorders in China: a cross-sectional epidemiological study. The Lancet Psychiatry. 2019;**6**(3):211-24.

3. Zhou M, Wang H, Zeng X, et al. Mortality, morbidity, and risk factors in China and its provinces, 1990–2017: a systematic analysis for the Global Burden of Disease Study 2017[J]. The Lancet, 2019, **394**(10204): 1145-1158.

4. Liu Y, Liu X, Wen H, et al. Risk behavior in patients with severe mental disorders: a prospective study of 121,830 patients managed in rural households of western China. BMC PSYCHIATRY. 2018;**18**(1):134.

5. Liang D, Mays VM, Hwang W. Integrated mental health services in China: challenges and planning for the future. HEALTH POLICY PLANN. 2018;**33**(1):107-22.

6. SCPRC (State Council of the People's Republic of China). Release of Management for Reporting the Incidence of Severe Mental Disorders (Trial); 2013.

7. NADPC (National administration of disease prevention and control). An integrated group of joint prevention and control mechanisms for the outbreak of new coronavirus pneumonia of the state council of China. Notice on Strengthening the Treatment and Management of Patients with Severe Mental Disorders during the Outbreak of the New Coronary Pneumonia (in Chinese); 2021.

8. Tan W, Lin H, Lei B, et al. The psychosis analysis in real-world on a cohort of large-scale patients with schizophrenia. BMC MED INFORM DECIS. 2020;**20**(3):132.

9. Vos T, Lim SS, Abbafati C, et al. Global burden of 369 diseases and injuries in 204 countries and territories, 1990–2019: a systematic analysis for the Global Burden of Disease Study 2019. The Lancet. 2020;**396**(10258):1204-22.

10. Flaxman AD, Vos DT, Murray CJ. An integrative meta-regression framework for descriptive epidemiology: University of Washington Press; 2015.

11. Keely C, Reed H, Waldman R. Understanding mortality patterns in complex humanitarian emergencies. Forced Migration and Mortality. National Academies Press, Washington, DC. 2001.

12. Berland J, Bogey C, Bailly C. Low-dissipation and low-dispersion fourth-order Runge-Kutta algorithm. COMPUT FLUIDS. 2006;**35**(10):1459-63.

13. Kauermann G, Opsomer JD. Data-driven selection of the spline dimension in penalized spline regression. BIOMETRIKA. 2011;**98**(1):225-30.

14. Wood SN. Generalized additive models: an introduction with R: CRC Press; 2017.

15. Vos T, Flaxman AD, Naghavi M, et al. Years lived with disability (YLDs) for 1160 sequelae of 289 diseases and injuries 1990-2010: a systematic analysis for the Global Burden of Disease Study 2010. The Lancet. 2012;**380**(9859):2163-96.

16. Gouda HN, Charlson F, Sorsdahl K, et al. Burden of non-communicable diseases in sub-Saharan Africa, 1990-2017: results from the Global Burden of Disease Study 2017. LANCET GLOB HEALTH. 2019;**7**(10):e1375-87.

17. Vos T, Abajobir AA, Abate KH, et al. Global, regional, and national incidence, prevalence, and years lived with disability for 328 diseases and injuries for 195 countries, 1990-2016: a systematic analysis for the Global Burden of Disease Study 2016. The Lancet. 2017;**390**(10100):1211-59.

18. Phanthunane P, Vos T, Whiteford H, Bertram M, Udomratn P. Schizophrenia in Thailand: prevalence and burden of disease. POPUL HEALTH METR. 2010;**8**(1):24.

19. Charlson FJ, Baxter AJ, Cheng HG, Shidhaye R, Whiteford HA. The burden of mental, neurological, and substance use disorders in China and India: a systematic analysis of community representative epidemiological studies. LANCET. 2016;**388**(10042):376-89.

20. Fullman N, Barber RM, Abajobir AA, et al. Measuring progress and projecting attainment on the basis of past trends of the health-related Sustainable Development Goals in 188 countries: an analysis from the Global Burden of Disease Study 2016. The Lancet. 2017;**390**(10100):1423-59.

21. Lim SS, Fullman N, Murray CJ, Mason-Jones AJ. Measuring the health-related Sustainable Development Goals in 188 countries: a baseline analysis from the Global Burden of Disease Study 2015. The Lancet. 2016:1-38.

22. Van der Loo M, van der Laan J, Team RC, Logan N, Muir C. Package ‘stringdist’. 2020.

23. Van der Loo MP. The stringdist package for approximate string matching. The R Journal. 2014;**6**(1):111-22.

24. Dreßler K, Ngonga Ngomo A. On the efficient execution of bounded jaro-winkler distances. SEMANT WEB. 2017;**8**(2):185-96.

25. Lin X, Liao Y, Hao Y. The burden of cardio-cerebrovascular disease and lung cancer attributable to PM2.5 for 2009, Guangzhou: a retrospective population-based study. INT J ENVIRON HEAL R. 2018:1-11.

26. Norman R, Cairncross E, Witi J, Bradshaw D. Estimating the burden of disease attributable to urban outdoor air pollution in South Africa in 2000. S Afr Med J. 2007;**97**(8 Pt 2):782-90.

27. Bonadiman CSC, Passos VMDA, Mooney M, Naghavi M, Melo APS. The Burden of disease attributable to mental and substance use disorders in Brazil: Global Burden of Disease Study, 1990 and 2015. Revista brasileira de epidemiologia. 2017;20Suppl 01(Suppl 01):191-204.

28. Murray CJL, Aravkin AY, Zheng P, et al. Global burden of 87 risk factors in 204 countries and territories, 1990-2019: a systematic analysis for the Global Burden of Disease Study 2019. The Lancet. 2020;**396**(10258):1223-49.

29. Sagar R, Dandona R, Gururaj G, et al. The burden of mental disorders across the states of India: the Global Burden of Disease Study 1990-2017. LANCET PSYCHIAT. 2020;**7**(2):148-61.

30. Roser M. Human development index (HDI). Our World in Data. 2014.

31. UNDP (UNITED NATIONS DEVELOPMENT PROGRAMME). Human Development Index (HDI). Human Development Reports; 2021.

32. GDSTATS (Guangdong Provincial Bureau of Statistics). Guangdong Statistical Yearbook (2010-2020); 2020.

33. Kyu HH, Abate D, Abate KH, et al. Global, regional, and national disability-adjusted life-years (DALYs) for 359 diseases and injuries and healthy life expectancy (HALE) for 195 countries and territories, 1990-2017: a systematic analysis for the Global Burden of Disease Study 2017. The Lancet. 2018;**392**(10159):1859-922.

34. Bogey C, Bailly C. A family of low dispersive and low dissipative explicit schemes for flow and noise computations. J COMPUT PHYS. 2004;**194**(1):194-214.

35. Hu FQ, Hussaini MY, Manthey JL. Low-dissipation and low-dispersion Runge-Kutta schemes for computational acoustics. J COMPUT PHYS. 1996;**124**(1):177-91.

36. Barendregt JJ, Van Oortmarssen GJ, Vos T, Murray CJ. A generic model for the assessment of disease epidemiology: the computational basis of DisMod II. POPUL HEALTH METR. 2003;**1**(1):4.

37. IHME. GitHub - ihmeuw/dismod_mr: An Integrative Metaregression Framework for Descriptive Epidemiology; 2021.

38. Lin X, Liao Y, Hao Y. The burden associated with ambient PM2.5 and meteorological factors in Guangzhou, China, 2012-2016: A generalized additive modeling of temporal years of life lost. CHEMOSPHERE. 2018;**212**:705-14.

39. Clements MS. Lung cancer rate predictions using generalized additive models. BIOSTATISTICS. 2005;**6**(4):576-89.

40. Lin X, Bloom MS, Du Z, Hao Y. Trends in disability-adjusted life years of lung cancer among women from 2004 to 2030 in Guangzhou, China: A population-based study. CANCER EPIDEMIOL. 2019;**63**:101586.

41. Murray CJ, Lopez AD, Jamison DT. The global burden of disease in 1990: summary results, sensitivity analysis and future directions. Bull World Health Organ. 1994;**72**(3):495-509.

42. WHO (World Health Organization). World health statistics 2010. WHO; 2010.

43. Bland RC, Newman SC, Orn H. Age and remission of psychiatric disorders. Can J Psychiatry. 1997;**42**(7):722-9.

44. Préville M, Boyer R, Vasiliadis H, et al. Persistence and remission of psychiatric disorders in the Quebec older adult population. The Canadian Journal of Psychiatry. 2010;**55**(8):514-22.
